# Phevalin (aureusimine B)Production by *Staphylococcus aureus* Biofilm and Impacts on Human Keratinocyte Gene Expression

**DOI:** 10.1371/journal.pone.0040973

**Published:** 2012-07-13

**Authors:** Patrick R. Secor, Laura K. Jennings, Garth A. James, Kelly R. Kirker, Elinor deLancey Pulcini, Kate McInnerney, Robin Gerlach, Tom Livinghouse, Jonathan K. Hilmer, Brian Bothner, Philip Fleckman, John E. Olerud, Philip S. Stewart

**Affiliations:** 1 Center for Biofilm Engineering, Montana State University, Bozeman, Montana, United States of America; 2 Department of Chemistry and Biochemistry, Montana State University, Bozeman, Montana, United States of America; 3 Functional Genomics Core Facility, Montana State University, Bozeman, Montana, United States of America; 4 Division of Dermatology, University of Washington, Seattle, Washington, United States of America; The Methodist Hospital Research Institute, United States of America

## Abstract

*Staphylococcus aureus* biofilms are associated with chronic skin infections and are orders of magnitude more resistant to antimicrobials and host responses. *S. aureus* contains conserved nonribosomal peptide synthetases that produce the cyclic dipeptides tyrvalin and phevalin (aureusimine A and B, respectively). The biological function of these compounds has been speculated to be involved in virulence factor gene expression in *S. aureus*, protease inhibition in eukaryotic cells, and interspecies bacterial communication. However, the exact biological role of these compounds is unknown. Here, we report that *S. aureus* biofilms produce greater amounts of phevalin than their planktonic counterparts. Phevalin had no obvious impact on the extracellular metabolome of *S. aureus* as measured by high-performance liquid chromatography-mass spectrometry and nuclear magnetic resonance. When administered to human keratinocytes, phevalin had a modest effect on gene expression. However, conditioned medium from *S. aureus* spiked with phevalin amplified differences in keratinocyte gene expression compared to conditioned medium alone. Phevalin may be exploited as potential biomarker and/or therapeutic target for chronic, *S. aureus* biofilm-based infections.

## Introduction

*Staphylococcus aureus* is an important human pathogen responsible for nosocomial and community-acquired infections associated with high morbidity and mortality [1]. Central to *S. aureus* pathogenicity is the formation of biofilms which are associated with chronic skin ulcers [2]. Biofilms exhibit unique phenotypic characteristics relative to planktonic bacteria such as increased resistance to antibiotics and host immune responses [3]. *S. aureus* has evolved mechanisms to fine-tune pathogenesis. Examples include the production of small molecules that regulate phenotypic changes in the pathogen (e.g. quorum sensing) and molecules that act directly on the host (e.g. virulence factors).

Recently, the production of the non-antibiotic pyrazinones tyrvalin, phevalin (also known as aureusimine A and B, respectively), and leuvalin were described for *S. aureus* [4], [5]. The pyrazinones are gene products of the *pzn* (*aus*) gene cluster that encodes a highly conserved nonribosomal peptide synthetase. Since the *pzn* gene cluster is highly conserved, the pyrazinones likely have an important biological function. Tyrvalin and phevalin were described as regulators of virulence factor gene expression in *S. aureus* [4]. However, it was later determined that an unintended mutation in the *sae* operon was responsible for the observed involvement of these dipeptides in virulence factor gene expression [6]. A clarification was recently published regarding the association of the pyrazinones with virulence factor gene expression in *S. aureus* [7]. In it, the authors suggest that the pyrazinones may direct a metabolic switch regulating electron transfer and redox signaling. The biological significance of these compounds remains to be elucidated.

**Figure 1:**
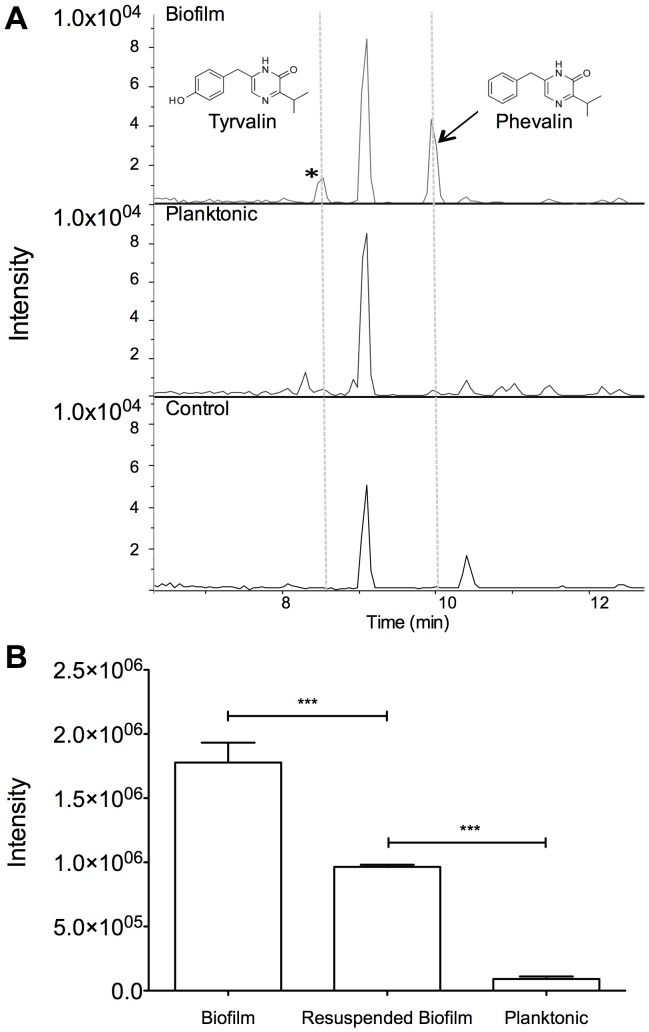
*S. aureus* biofilms produce more phevalin than their planktonic counterparts. (A) HPLC-MS analysis of organic extracts from *S. aureus* biofilm, planktonic, and growth medium control revealed that biofilms produce more phevalin (aureusimine B) than planktonic cultures (arrow). A compound that is likely tyrvalin (aureusimine A) was also present at higher levels in the biofilm (*). (B) Phevalin production was detected directly in samples without prior organic extraction. Samples were normalized to cell density (optical density, 600 nm, OD_600_) in biofilm (OD_600_ 0.9), resuspended biofilm (OD_600_ 1.4), and planktonic cultures (OD_600_ 0.66). Data represent means ± SEM, n = 3, ***p<0.001.

Phevalin was originally discovered in a soil actinomycete and was reported to exhibit calpain inhibitor activity [8]. Calpains are intracellular heterodimeric cysteine proteases that participate in a variety of eukaryotic cellular processes including cell motility, apoptosis, and cell cycle progression [9]. In mammals, two ubiquitous isoforms of calpain exist, μ-calpain and m-calpain, and both are present in the epidermis [10]. In addition to µ-calpain and m-calpain, twelve additional isoforms of calpain are encoded by the human genome [11]. The calpain inhibitor activity of phevalin on μ-calpain was non-existent [12] suggesting that another isoform of calpain may be the target of phevalin.

**Table 1:** Phevalin production in various strains of bacteria as detected by SRM HPLC-MS.

Organism	Phevalin detected (X) or not detected (O)
*Acinetobacter baumannii* clinical *isolate*	O
*Actinomyces naeslundii* ATCC 19039	O
*Enterococcus faecalis* clinical isolate	O
*Escherichia coli* ATCC 25922	O
*Porphyromonas gingivalis* clinical isolate	O
*Pseudomonas aeruginosa* ATCC 27853	O
*Pseudomonas aeruginosa* clinical isolate	O
*Pseudomonas pneumonia* 6303	O
*S. aureus* ATCC 29213	X
*S. aureus* ATCC 33591	X
*S. aureus* clinical isolate	X
*S. aureus* clinical isolate 10943 (used in this study)	X
*S. aureus* strain ALC2085	X
*Streptococcus mutans* ATCC 33535	X
*Streptococcus oralis* ATCC 10557	O
TSB control	O
Uncharacterized oral community	X
*Veillonella parvula* ATCC 17745	O

As part of our effort to characterize the extracellular metabolome of *S. aureus* biofilms, we set out to identify molecules associated with the biofilm phenotype and investigate how these molecules may influence the host/pathogen interface. One metabolite in particular, phevalin, was produced in greater quantities by *S. aureus* biofilms relative to their planktonic counterparts. Here we report that phevalin by itself has only modest effects on human keratinocytes (HKs) or *S. aureus*, but combined with other yet to be identified soluble bacterial products, HK gene expression was impacted.

**Table 2:** After the addition of 1 µM or 10 µM phevalin to HKs, only 24 genes were significantly regulated (±2 fold in any one condition, p<0.05) relative to control cells.

Gene Symbol	OWA pval	FC 1 uM v Control	FC 10 uM v Control
*TP63*	2.97E-02	3.15	8.28
*N4BP2L2*	3.70E-02	2.65	1.30
*EXOC5*	4.45E-02	2.19	2.55
*TRA2A*	1.53E-02	2.17	−1.09
*GNS*	2.95E-02	2.04	2.33
*CUL4B*	1.64E-02	1.98	2.12
*CPNE3*	3.38E-02	1.42	2.90
*C13orf15*	1.61E-04	−1.26	−2.05
*BAT2L2*	4.10E-02	−1.38	−2.39
*FERMT2*	1.79E-02	−1.69	−2.00
*SLC7A1*	1.85E-02	−1.79	−2.02
*DSC2*	1.91E-02	−1.80	−2.14
*EZR*	3.26E-03	−1.82	−2.59
*CCND1*	2.45E-02	−1.82	−2.17
*SON*	3.41E-02	−1.89	−2.99
*CALD1*	1.50E-03	−1.91	−2.62
*TOP1*	1.21E-02	−1.99	−2.91
*IQGAP1*	1.26E-02	−2.07	−2.49
*KLF7*	6.58E-03	−2.09	−1.87
*MICAL2*	2.02E-02	−2.10	−1.69
*KRAS*	4.29E-02	−2.24	−2.64
*EGR1*	2.00E-02	−2.55	−1.27
*TAGLN*	3.80E-03	−3.73	−4.48
*CXCL14*	1.25E-02	−4.56	−4.45

## Results

### Phevalin Production by S. aureus Biofilms

Initial analysis of chloroform-extracted bacteria-conditioned medium by high-performance liquid chromatography-mass spectrometry (HPLC-MS) revealed two predominant peaks in biofilm samples (Figure 1A). These peaks had m/z values that matched the reported m/z values for tyrvalin (245.1285) and phevalin (229.1335) [4], [5]. Phevalin in spent culture medium was confirmed by exact mass, retention time, and tandem mass spectrometry compared to synthetic phevalin (Figure S1A-D). The fragmentation pattern of a putative tyrvalin in biofilm samples was consistent with that expected for tyrvalin (Figure S1E).

**Figure 2:**
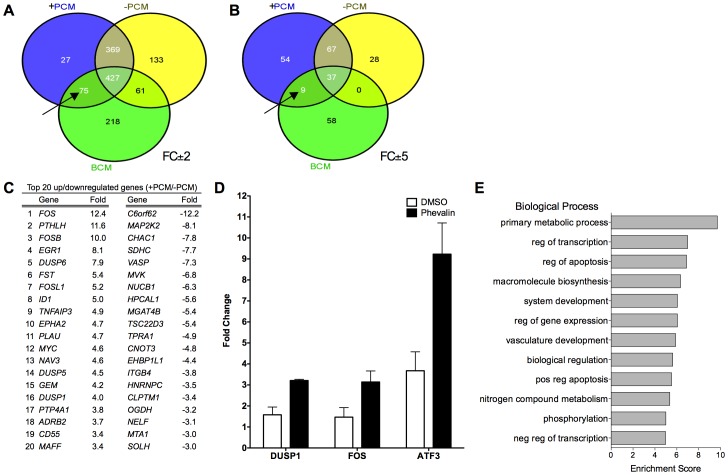
Conditioned medium from *S. aureus* cultures with or without additional phevalin induces differential gene expression in HKs. Significant (p<0.05) transcripts regulated ±2 fold in any one condition relative to controls. Transcripts shared between HKs treated with BCM, +PCM, and –PCM are shown at ±2 and ±5 fold change cutoffs (A and B, respectively). HKs treated with BCM shared more transcripts with +PCM treated HKs than –PCM treated HKs (arrows). Transcripts shared between –PCM and BCM had modest fold changes as no transcripts were shared above the ±5 FC cutoff. (C) The top 20 upregulated and downregulated genes (p<0.05) in +PCM treated HKs relative to –PCM treated HKs are listed. For a complete list of significantly regulated genes, see Table S1. (D) Selected genes were confirmed by RT-qPCR. The fold change relative to a GAPDH normalizer is indicated (p<0.05 for all comparing DMSO to phevalin). (E) Functional annotation clustering of microarray data revealed significantly (Benjamini p<0.01) enriched biological processes in +PCM treated HKs.

Phevalin was analyzed in cultures inoculated with either planktonic overnight (low biomass) or resuspended biofilms (high biomass) to control for differing growth phases and cell densities. *S. aureus* biofilms produced more phevalin than stationary phase cultures or cultures inoculated with resuspended biofilms (Figure 1B). Phevalin production was also observed in spent medium from *S. aureus* biofilms grown in a colony drip flow reactor under continuous flow as previously described [13] with increasing amounts of phevalin detected each day over five days (data not shown). Several strains of bacteria were tested for the presence of phevalin in spent culture medium (Table 1). Phevalin was detected in all strains of *S. aureus* tested (MRSA and methicillin-susceptible strains), *Streptococcus mutans* and in an undefined oral community derived from human saliva. Phevalin production was not detected in spent medium from any Gram-negative bacteria tested.

### Influence of Phevalin on HK Gene Expression

Since phevalin was first identified as a calpain inhibitor [8], we investigated whether or not phevalin had an impact on HKs. In response to 1 µM or 10 µM phevalin, 24 genes were found to be significantly regulated ±2 fold (p<0.05) in any one condition relative to control cells (Table 2). Of those genes, the majority had modest fold changes between ±2 and ±3. The most highly upregulated gene (+8.28 fold) was the transcription factor *TP63* (p63), which is part of the family of transcription factors that includes p73 and p57 which function to induce cell cycle arrest and apoptosis [14]. Calpains regulate the stability of p73 [15], but it remains to be determined if p63 is regulated in the same manner.

Since phevalin was produced in greater quantities by *S. aureus* biofilm, we hypothesized that exposure of planktonic *S. aureus* to increased amounts of phevalin would induce the production of extracellular molecules normally produced by biofilms. We observed that substantial differences exist in the composition of *S. aureus* biofilm-conditioned medium (BCM) and planktonic-conditioned medium (PCM) (Jennings et al., in preparation). We have also observed that *S. aureus* BCM and PCM induce differential gene expression in HKs [16]. Given that phevalin by itself did not induce a substantially different transcriptional profile in HKs, we sought to use HK gene expression as a diagnostic tool to screen spent medium from planktonic *S. aureus* exposed to increased amounts of phevalin for unique or biofilm-associated HK responses.

PCM from *S. aureus* cultures exposed to phevalin (+PCM) or DMSO (–PCM) was harvested. The transcriptomes of HKs exposed to +PCM or –PCM were analyzed. For comparison, HKs exposed to DMSO (control) or BCM spiked with DMSO were also analyzed. A large number of genes (1531) were significantly regulated ±2 fold (p<0.05) in any one condition, relative to control cells (Table S1). Overall, +PCM and –PCM treated HKs were more similar in their transcriptional profiles compared to BCM treated HKs (Figure 2A and 2B). However, +PCM treated HKs shared more significantly regulated transcripts with BCM treated HKs than –PCM treated HKs indicating that +PCM induced a slightly more biofilm-like response from HKs than –PCM.

Comparison of transcriptional profiles of +PCM treated HKs to –PCM treated HKs revealed164 genes that were significantly regulated ±2 fold (p<0.05) (Table S2). The top 20 upregulated or downregulated genes in +PCM treated HKs relative to –PCM treated HKs are listed in Figure 2C. Several of the most highly upregulated genes encoded transcription factors, notably members of the FOS family. Members of the FOS family, along with ATF and JUN family members, form various heterodimers creating the activator protein-1 (AP-1) complex [17]. *ATF3* was also upregulated 2.1 fold in +PCM treated HKs relative to –PCM treated cells (Table S2). AP-1 regulates the transcription of a variety of genes relating to inflammation, pathology, and homeostasis in the skin and is activated by mitogen-activated protein kinase (MAPK) cascades [18]. Dual specificity phosphatases (DUSPs) are negative regulators of MAPK signaling. Several DUSP family members were upregulated in +PCM treated HKs relative to –PCM treated HKs. DUSP, FOS, and ATF family members are all intimately associated with MAPK signaling cascades [19]. The upregulation of *DUSP1*, *ATF3*, and *FOS* in +PCM treated HKs was confirmed by reverse transcription-quantitative polymerase chain reaction (RT-qPCR) (Figure 2D).

**Figure 3:**
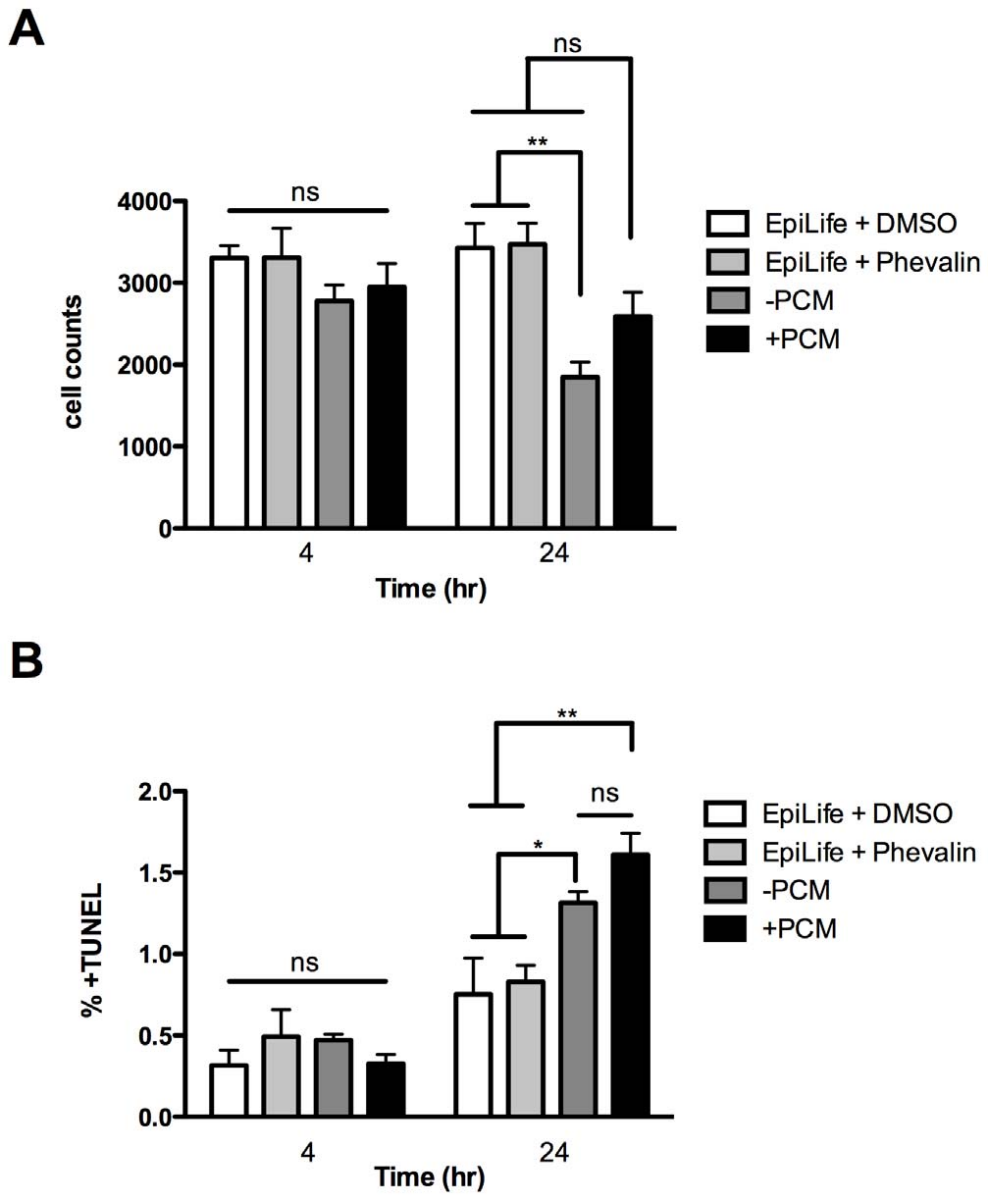
Phevalin does not induce apoptosis in HKs. (A) Cell counts after 4 or 24 hours of exposure to phevalin, -PCM, or +PCM. (B) Percent cells staining positive for TUNEL after 4 or 24 hours of exposure to phevalin, -PCM, or +PCM. Data represent ± SEM, n = 6, *p<0.05, ** p<0.01, ns, not significant, p>0.05.

Since functional annotation clustering analysis revealed biological processes related to apoptosis (Figure 2E) and calpains are regulators of apoptosis [20], late stage apoptosis was investigated by terminal deoxynucleotidyl transferase dUTP nick end labeling (TUNEL). Induction of apoptosis was higher in +PCM and –PCM compared to controls (Figure 3). However, induction of late stage apoptosis in HKs exposed to +PCM was not statistically significantly different from HKs exposed to –PCM.

### Observed Impact of Phevalin on the Extracellular Proteome/metabolome of S. aureus is Minimal

**Figure 4:**
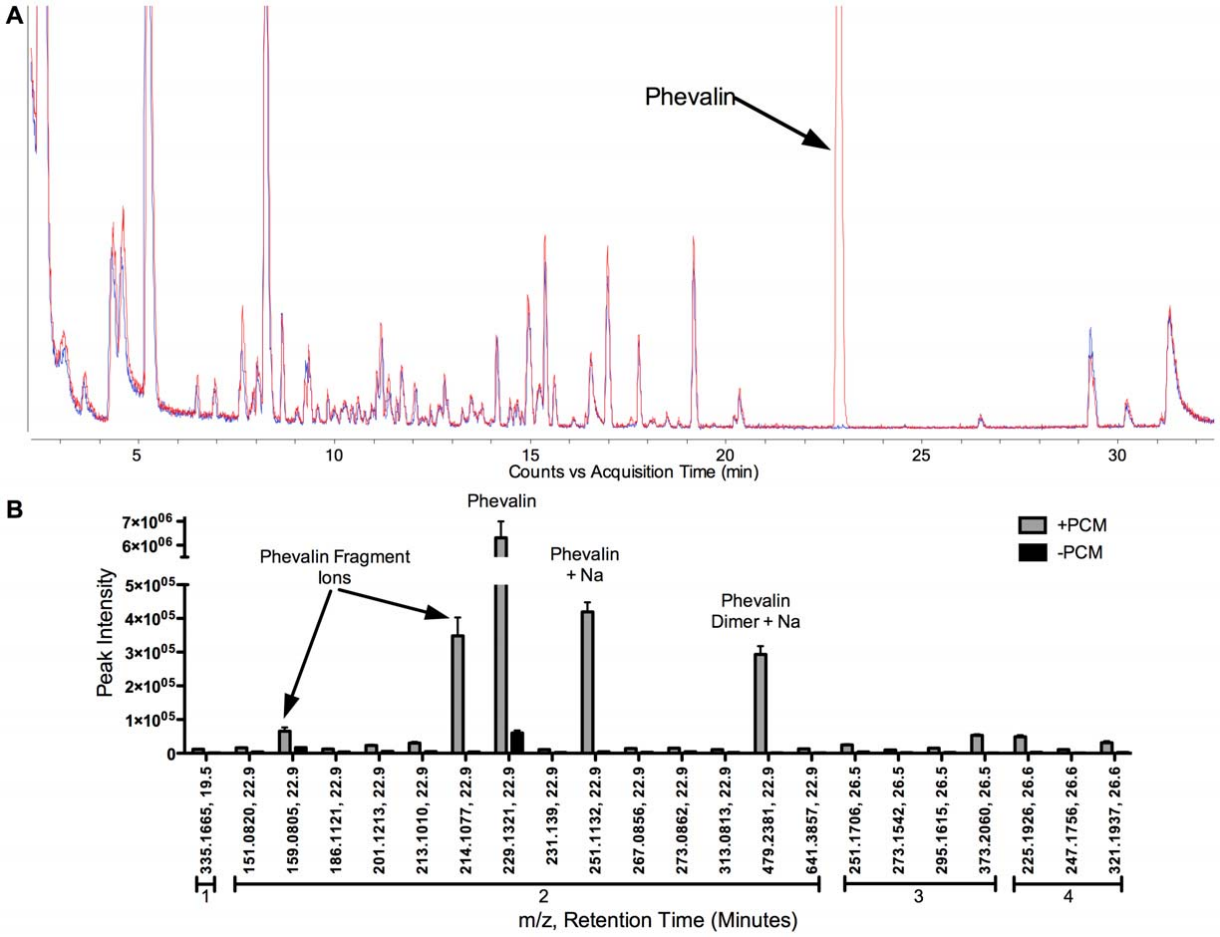
Increased amounts of phevalin did not impact the extracellular metabolome of *S. aureus* as detected by HPLC-MS. (A) Overlays of representative HPLC-MS base-peak chromatograms of +PCM (red) and –PCM (blue) revealed no substantial differences with the exception of phevalin (arrow). (B) XCMS analysis identified 22 features significantly (p<0.05) regulated ±2 fold in +PCM relative to –PCM. Those features grouped by RT into four groups (numbered bars). Group 2 contained phevalin, its fragment ions, and various adducts. Groups 1, 3, and 4 contained trace features associated with compounds used during the synthesis of phevalin. Data represent means ± SEM, n = 3.

We sought to determine if phevalin induced the production of extracellular molecules in *S. aureus* that may be responsible for the observed transcriptional differences in HKs treated with +PCM or –PCM. Spiked-in phevalin had no impact on the growth of *S. aureus* (data not shown). Levels of phevalin were monitored by selected reaction monitoring (SRM) HPLC-MS at two-hour intervals post spike-in. No appreciable degradation of phevalin was observed six hours post phevalin spike-in (data not shown). Visual inspection of HPLC-MS chromatograms of +PCM and –PCM revealed no obvious differences (Figure 4A). HPLC-MS data were further analyzed by XCMS, an algorithm designed to analyze LC-MS datasets by nonlinear-retention-time alignment, feature detection and matching, and automatic integration and extraction of peak intensities [21]. Only 22 features with a fold change of ±2, p<0.05, were identified in +PCM and –PCM (Figure 4B). Of those 22 features, four groups with different retention times (RT) were apparent, suggesting the presence of only four different compounds and their various adducts. Of those four RT groups, one contained phevalin, its fragment ions, and various adducts. Comparing features present in unpurified phevalin with HPLC purified phevalin revealed that the other RT groups consisted of trace compounds used in the synthesis of phevalin. HPLC-MS analysis estimated that the phevalin used in this study was ∼98% pure.

**Figure 5:**
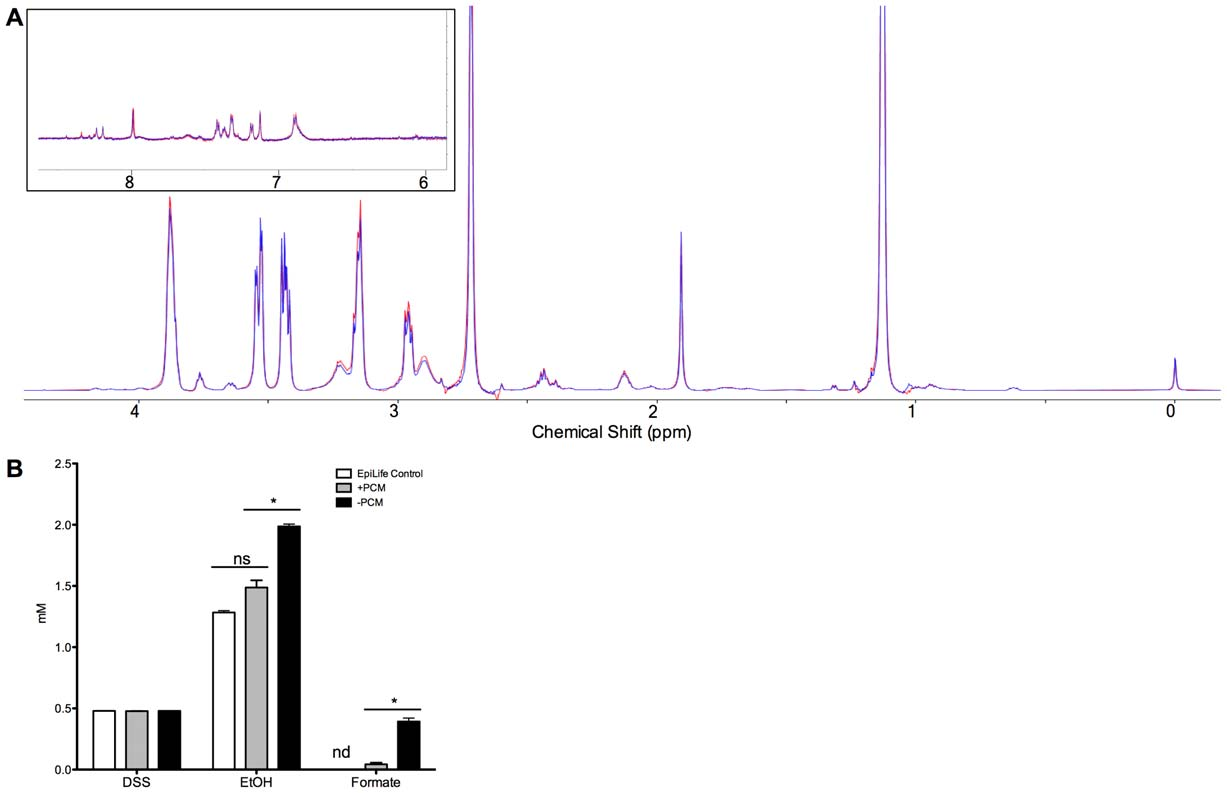
Analysis of +PCM and –PCM by NMR revealed minimal differences in metabolite composition. (A) Representative raw NMR spectra of +PCM (red) and –PCM (blue) did not show any major differences in metabolite compositions. (B) Analysis of NMR spectra by Chenomx revealed only two compounds, ethanol and formate, that were produced in higher quantities in –PCM relative to +PCM. DSS is the chemical shape indicator. Data represent means ± SEM, n = 3, *p<0.02. ns, not significant, nd, not detected.

Analysis of +PCM and –PCM by nuclear magnetic resonance (NMR) revealed minimal differences in sample spectra (Figure 5A). Selected metabolites were quantified by comparison to DSS peak area using Chenomx software. Ethanol and formate were the only metabolites identified with significant differences (p<0.05) in concentration between +PCM and –PCM samples (Figure 5B). Extracellular proteomic analysis using 1D SDS-PAGE analysis revealed no obvious differences in protein banding patterns between +PCM and –PCM (Figure S2).

## Discussion

In this study, we show that *S. aureus* biofilms produce increased amounts of the nonribosomal peptide phevalin relative to their planktonic counterparts. *S. aureus* biofilms also produce a putative tyrvalin. The effect of phevalin on the *S. aureus* extracellular proteome and metabolome was surprisingly small, which led us to explore possible effects of phevalin in the host. Overall, the HK transcriptional profile induced by +PCM is consistent with the regulation of MAPK/AP-1 dependent signaling cascades. The effect of phevalin on HK gene expression was significantly compounded by the presence of soluble bacterial products, possibly because those products activate signaling pathways not active in resting HKs. The deranged activation of MAPK/AP-1 signaling cascades would likely impact several biological processes relating to wound healing including apoptosis, proliferation, and cell migration.

Functional annotation clustering of transcriptomic data indicated that several biological processes were enriched in +PCM treated HKs, including apoptosis. We have previously shown that BCM induces apoptosis in HKs while PCM does not [22]. However, induction of late stage apoptosis, as indicated by a TUNEL assay, was not statistically significant in HKs exposed to +PCM compared to –PCM, possibly because phevalin induces earlier stages of apoptotic programs which can be aborted.

Originally, we hypothesized that planktonic *S. aureus* exposed to increased levels of phevalin would synthesize soluble products normally produced by biofilms. However, minimal differences between the extracellular metabolome and proteome of *S. aureus* exposed to phevalin were observed. Although it is impossible to obtain complete coverage of the metabolome, two complementary metabolite-profiling techniques were used [23]. NMR was used to profile abundant, hydrophilic compounds while reverse phase HPLC-MS was utilized for a more sensitive analysis of hydrophobic molecules. It is possible that small differences in ethanol and formate in +PCM and –PCM partially influenced gene expression in HKs. At high concentrations (10 mM) formate can impact the viability of oral epithelial cells [24]. Concentrations of ethanol above 171 mM have toxic effects on HKs in vitro [25]. However, at the low levels of formate and ethanol observed in this study, it is more likely that the observed differences in HK gene expression could be attributed to the large difference in phevalin in –PCM and +PCM. Analysis of +PCM and –PCM by 1D SDS-PAGE did not reveal any obvious differences in protein banding patterns. However, a more in depth proteomic analysis may reveal proteins that could potentially contribute to the observed HK transcriptional response.

The biological function of phevalin is of great interest. Sun et al. were not able to link phevalin to virulence in a murine infection model under the conditions tested [6]. No significant difference in weight loss was observed between mice injected with 10^7^ cfu wild type *S. aureus* or the Δ*aus* mutant not capable of producing phevalin. In bacteria, nonribosomal peptides can have numerous functions including motility, antimicrobial activity, and biofilm formation or disruption [26]. The original description of phevalin stated that it inhibited calpain proteolytic activity, although a specific isoform of calpain was not isolated and tested [8]. It is likely that phevalin is more or less potent against the various isoforms of calpain. Several genes with links to calpain activity were identified by microarray analysis of +PCM and –PCM treated HKs. A member of the calpain family (calpain 15 or *SOLH*) was downregulated in +PCM treated HKs. *SOLH* is involved in the development of the visual system [27], but has no known function in the skin. The gene encoding the transcription factor MYC, which was upregulated in +PCM treated HKs, regulates calpain-mediated apoptosis [28]. Integrin β4 (*ITGB4*) was downregulated in +PCM treated HKs. Integrin β4 mediates cell-cell and cell-substratum adhesions and is degraded by calpain [29]. Lastly, it is well established that AP-1 constituents, particularly FOS, are degraded by calpains [30].

In addition to degrading AP-1 family members, the calpains interact directly with MAPK cascades. In the epidermis, the MAPK member ERK directly phosphorylates and activates m-calpain, which destabilizes cell-substratum adhesions, enabling cell migration [31]. MAPK dependent m-calpain activation by staphylococcal protein A destabilizes cell-cell junctions facilitating staphylococcal transmigration through the epithelium [32]. Bacterial products that stimulate MAPK dependent activation of calpain promote the recruitment of polymorphonuclear leukocytes (PMNs) into infected airways [33]. Inhibition of calpain nearly completely blocked the migration of PMNs across the airway epithelium. Bacterial manipulation of MAPK/calpain dependent responses such as leukocyte recruitment and epithelial integrity would have dramatic impacts in staphylococcal infections. As a chronic and localized biofilm infection develops, an increase in the production of phevalin may lead to deranged MAPK/calpain activity, inappropriate regulation of epithelial integrity or epithelialization, altered leukocyte recruitment, and ultimately the disruption of normal wound healing processes.

Our results demonstrate that *S. aureus* biofilms produce increased amounts of phevalin. Phevalin, in the presence of other soluble bacterial factors, induced differential gene expression in HKs relative to soluble bacterial factors alone. While the specific biological activities of the pyrazinones remain to be elucidated, phevalin may play a potential role in the pathogenesis of *S. aureus* skin infections. As such, phevalin may be a potential therapeutic target in *S. aureus* skin infections, particularly biofilm-based diseases. The detection of increasing amounts of phevalin in infected tissues may be diagnostic for the establishment of a biofilm-based infection and could potentially be used to guide treatment strategies.

## Materials and Methods

### Culture Conditions

The spontaneously immortalized human HaCaT keratinocyte cell line was used. HKs were maintained in EpiLife keratinocyte growth medium (Invitrogen, Carlsbad, CA) supplemented with human keratinocyte growth supplement (HKGS; Invitrogen) hereafter referred to as Epi. HKs were cultured in a humidified 5% CO_2_ atmosphere at 37°C. A clinical isolate of *S. aureus* (Southwest Regional Wound Care isolate # 10943, Lubbock, TX) grown in Epi was used in all experiments except where noted otherwise. Colony biofilms were grown as described previously [22]. Briefly, biofilms were grown on tissue culture inserts (35 mm diameter, 0.2 µm pore size, Nalge Nunc International, Rochester, NY) placed into six-well plates with 2.1 ml Epi in each well. Mature biofilms (72 hours, biological triplicates) were placed in fresh Epi for an additional 24 hours. The conditioned growth medium was then collected and filter sterilized. Colony drip flow biofilms were grown as described previously [13]. Spent drip flow medium was collected once every 24 hours for five days. Planktonic cultures of *S. aureus* were maintained at 37°C with constant agitation in Epi. Resuspended biofilm cultures were prepared as described previously [16]. Briefly, 72 hour biofilms were resuspended in 2.1 ml Epi/insert and grown with constant agitation at 37°C for an additional 24 hours (biological triplicates).

### Synthesis of Phevalin

Synthesis of phevalin was performed as described previously [12]. Synthetic phevalin was purified by HPLC. Elution of phevalin was monitored by UV (322 nm) on an Agilent (Santa Clara, CA) 1200 series HPLC with a Phenomenex (Torrance, CA) Jupiter 4 µm proteo 90A 250×10mm column. Collected fractions were pooled, dried under nitrogen, and resuspended in endotoxin-free DMSO to a final concentration of 10 mM. A water blank was subjected to an identical purification protocol. Blank samples were collected, dried, and resuspended in DMSO. This DMSO was used as the vehicle control in all experiments.

### Sample Extraction

Bacteria listed in Table 1 were grown in 25 ml TSB at 37°C overnight with constant agitation. Bacteria were removed by centrifugation and the spent medium was filter sterilized. Two milliliters of spent medium were added to an equal volume of chloroform in glass tubes and vortexed. Spent medium from biofilm or planktonic cultures was also extracted into chloroform. Organic fractions were removed to fresh glass tubes and dried under a stream of filtered nitrogen. The dried material was resuspended in 100 µl of 20% DMSO in water. Samples were analyzed by SRM HPLC-MS as described below.

### Phevalin Spike

Phevalin was spiked into planktonic *S. aureus* cultures during mid-exponential phase growth (∼6 hours, 10 µM phevalin, 0.1% DMSO, +PCM) and the cultures were maintained for an additional 8 hours to reach a stationary growth phase. A control culture (–PCM) was grown under identical conditions using 0.1% DMSO as a vehicle control. Bacteria were removed from solution by centrifugation and samples were filter sterilized. Samples were analyzed by HPLC-MS, NMR, and 1D SDS-PAGE.

### HPLC-MS

Samples were separated on an Agilent 1290 uHPLC (Agilent Technologies, Santa Clara, CA) with a Phenomenex Kinetex C-18 column (150×2.1 mm with 2.6 µm particle size, Torrance, CA) at 40°C with a flow rate of 0.4 ml/min. Mobile phases used were A: water plus 0.1% formic acid and B: 95% acetonitrile plus 0.1% formic acid. A gradient elution program was used: 0−2 min 2% B, 2–5 min 2% B ramped to 95% B by 35 min and held for 5 min before returning to initial conditions and equilibrating for 10 min before injection of the next sample. An injection volume of 5 µl was used for organic extracts and 10 µl for non-extracted material. Ion detection was performed on an Agilent 6538 Q-TOF in positive mode. Mass spectrometry conditions were as follows: source voltage, 3.5 kV; skimmer voltage, 65V; dry gas flow, 10 l/min; and dry gas temperature, 200°C. XCMS was used for retention time correction and ion feature generation using default parameters [21]. SRM was employed to detect phevalin using the same instrumentation and mobile phases described above. Chromatographic separation was performed isocratically at 70% B on an Agilent SB-C18 1.8 µm 2.1×100mm column (Santa Clara, CA) at 40°C at 0.4 ml/min. Ion detection was performed in positive mode. A collision energy of 20 eV was used to follow the 229.13>214.11 transition.

### NMR

NMR metabolite profiling was conducted as follows: phosphate buffer (1 M, pH 6.8–7.0) in deuterium oxide containing 5 mM 2,2-dimethyl-2-silapentane-5-sulfonate sodium salt (DSS) as an internal standard, 0.2% sodium azide as a preservative, and 20 mM difluorotrimethylsilanylphosphonic acid (DFTMP) as a pH indicator was added as a 10% v/v spike to +PCM and –PCM samples. NMR spectra were acquired on a 600 MHz Bruker DRX solution NMR spectrometer using a 1-D NOESY pulse sequence. NMR spectra were phased, baseline corrected and overlaid using Chenomx NMR suite 7.0 software (Chenomx Inc. Edmonton, Canada). Selected metabolite concentrations were determined by comparison to DSS peak area.

### SDS-PAGE

One dimension SDS-PAGE analysis of proteins in +PCM and –PCM was performed as described previously [16]. Briefly, trichloroacetic acid precipitated proteins were re-suspended into loading buffer containing β-mercaptoethanol and boiled for 5 minutes. Samples were run on 12% acrylamide precast gels (BioRad, Hercules, CA) and stained with Sypro Ruby (Invitrogen, Carlsbad, CA).

### TUNEL

TUNEL (terminal deoxynucleotidyl transferase dUTP nick end labeling) staining was used to investigate apoptosis. HKs were cultured in 96-well plates (5000 cells/well) for two days. Once 80–90% confluence was reached, HK cultures were exposed to +PCM, **−**PCM, Epi +0.1% DMSO, or Epi +10 µM Phevalin (0.1% DMSO). After 4 and 24 hours of exposure, the conditioned medium was removed, and the cultures were fixed in 4% paraformaldehyde in PBS for 15 minutes at 37°C followed by three 5 minute washes in PBS. Ethanol (70%) was then added to the cultures, which were stored at −20°C until assayed. The APO-BrdU TUNEL Assay Kit (Invitrogen) was used, and the manufacturer’s staining protocol was adapted for fluorescence microscopy. All enzyme solutions were made in the same proportions suggested by the manufacturer, but were added directly to the culture plate. The cultures were then imaged using a Nikon Eclipse E800 epi-fluorescent microscope using a 10× objective, and the percentage of cells staining positive for TUNEL was enumerated. Control cultures were also stained and consisted of HK exposed to standard culture medium. Multiple cultures (n = 6) were used for all conditions.

### Microarray

HKs were grown to confluence in six well plates in Epi (biological triplicates for all conditions tested). Cells were treated with 1 ml of 0 µM (vehicle control), 1 µM, or 10 µM phevalin (all 0.1% DMSO). For the ±PCM experiment, HKs were treated with 1 ml of +PCM, 1 ml of –PCM, or 1 ml of BCM supplemented with 0.1% DMSO. Microarray analysis was performed as described previously [16]. Briefly, after four hours, the medium was removed and RNA was isolated using an RNeasy minikit (Qiagen, Valencia, CA). Total RNA (500 ng) was reverse transcribed, amplified, and biotin-labeled via in vitro transcription using the MessageAmp Premier kit (Applied Biosystems/Ambion, Austin, TX). The resulting cRNA was fragmented and hybridized to Affymetrix GeneChip Human Genome U133A 2.0 arrays (#900468, Affymetrix, Santa Clara, CA). Microarray data were analyzed using FlexArray version 1.4. Analysis of Variance (ANOVA) was performed to identify statistically significant differences among the different conditions. Data were submitted to Gene Expression Omnibus (GEO; NCBI) under accession GSE32920. The Database for Annotation, Visualization and Integrated Discovery’s (DAVID) functional annotation tool was used to cluster biological processes using high stringency [34], [35].

### Quantitative Polymerase Chain Reaction Analysis of Gene Expression

Reverse transcription-quantitative polymerase chain reaction (RT-qPCR) analysis on equivalent amounts of total RNA (500 ng) was performed using the QuantiTect Reverse Transcription Kit according to the manufacturer’s instructions (Qiagen, Valencia, CA). Primers used for qPCR reactions are shown in Table S3. PCR was carried out in a RotorGene 3000 thermal cycler (Corbett Life Science, Mortlake, NSW, Australia). Equivalent amounts of cDNA generated from RT reactions were used as a template for PCR using the QuantiFast SYBR Green PCR Kit (Qiagen, Valencia, CA). Reactions were performed in triplicate for each sample. The thermal profile for PCR consisted of an activation step of 95°C for 5 minutes, then 40 cycles of denaturation at 95°C for 10 seconds, followed by annealing and extension at 50°C for 30 seconds. For each sample, expression of target and marker genes was normalized to the expression of glyceraldehyde 3-phosphate dehydrogenase (GAPDH). Data are expressed as the fold change in expression (phevalin or DMSO vehicle control) relative to no treatment.

### Statistical Methods

The standard error of measurement (SEM) was calculated and data were analyzed by a two-tailed unpaired t-test using GraphPad Prism 5 software.

## Supporting Information

Figure S1 **HPLC-MS/MS confirms that phevalin is present in biological samples.** (A-D) The retention time (A, biological, and B, synthetic) and fragmentation patterns (C, biological, and D, synthetic) between the biological and synthetic molecule are identical. (E) Fragmentation pattern of putative tyrvalin produced by S. aureus biofilm. Two tyrvalin fragment ions have m/z values 16 units larger than corresponding fragment ions produced by phevalin (214.1079 and 230.1043; 159.089 and 175.0853). This is constant with the additional oxygen present in the tyrosine residue of tyrvalin.
(PDF)

Figure S2 **1D SDS-PAGE analysis of extracellular proteins in growth medium control and +PCM and −PCM.** No obvious differences were apparent between +PCM and **−**PCM. Gel stained with Sypro Ruby with a lower detection limit of 0.25–1 ng.
(PDF)

Table S1 **Genes significantly (p<0.05) regulated at least 2 fold in HKs treated with +PCM, −PCM, or BCM, relative to controls.**
(PDF)

Table S2 **Genes significantly (p<0.05) regulated at least 2 fold in HKs treated with +PCM relative to −PCM treated HKs.**
(PDF)

Table S3 **qPCR primer sequences.**
(PDF)
