# Correction: Phevalin (aureusimine B)Production by *Staphylococcus aureus* Biofilm and Impacts on Human Keratinocyte Gene Expression

**DOI:** 10.1371/annotation/0fc3d60c-8ae4-4f04-be17-d5d245b4eef5

**Published:** 2012-10-11

**Authors:** Patrick R. Secor, Laura K. Jennings, Garth A. James, Kelly R. Kirker, Elinor deLancey Pulcini, Kate McInnerney, Robin Gerlach, Tom Livinghouse, Jonathan K. Hilmer, Brian Bothner, Philip Fleckman, John E. Olerud, Philip S. Stewart

The following information is missing from the funding statement: LKJ was funded in part by the ARRA supplement grant to the CoBRE “Center for the Analysis of Cellular Mechanisms and Systems Biology” (3P20RR024237-02S1). Support for the NMR Facility at MSU was provided by the NIH Shared Instrumentation Grant program (grants # 1S10RR13878 and 1S10RR026659). 

