# Supplementary figures and images for: Phevalin (aureusimine B)Production by Staphylococcus aureus Biofilm and Impacts on Human Keratinocyte Gene Expression
Source: PLoS One. 2012 Jul 13;7(7):e40973. doi: 10.1371/journal.pone.0040973 (PMC3396627; doi:10.1371/journal.pone.0040973)

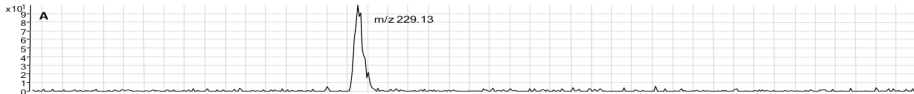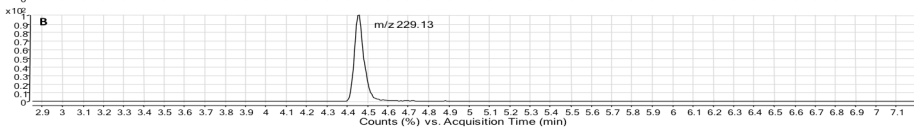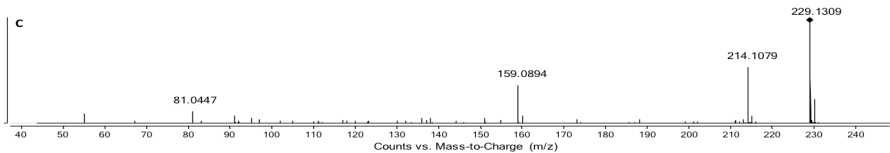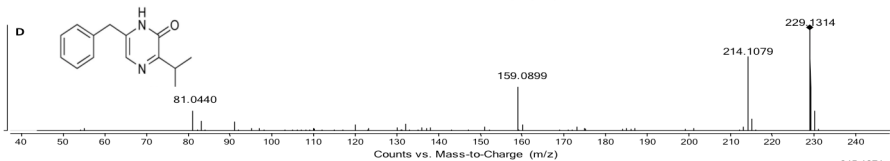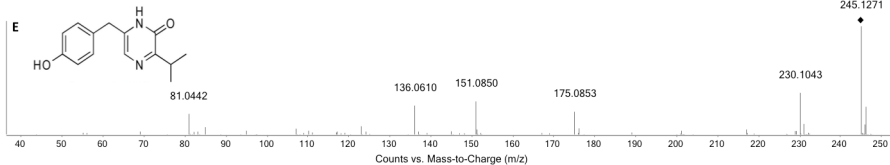

Supplement: Figure S1 — HPLC-MS/MS confirms that phevalin is present in biological samples. (A-D) The retention time (A, biological, and B, synthetic) and fragmentation patterns (C, biological, and D, synthetic) between the biological and synthetic molecule are identical. (E) Fragmentation pattern of putative tyrvalin produced by S. aureus biofilm. Two tyrvalin fragment ions have m/z values 16 units larger than corresponding fragment ions produced by phevalin (214.1079 and 230.1043; 159.089 and 175.0853). This is constant with the additional oxygen present in the tyrosine residue of tyrvalin. (PDF) [file pone.0040973.s001.pdf]

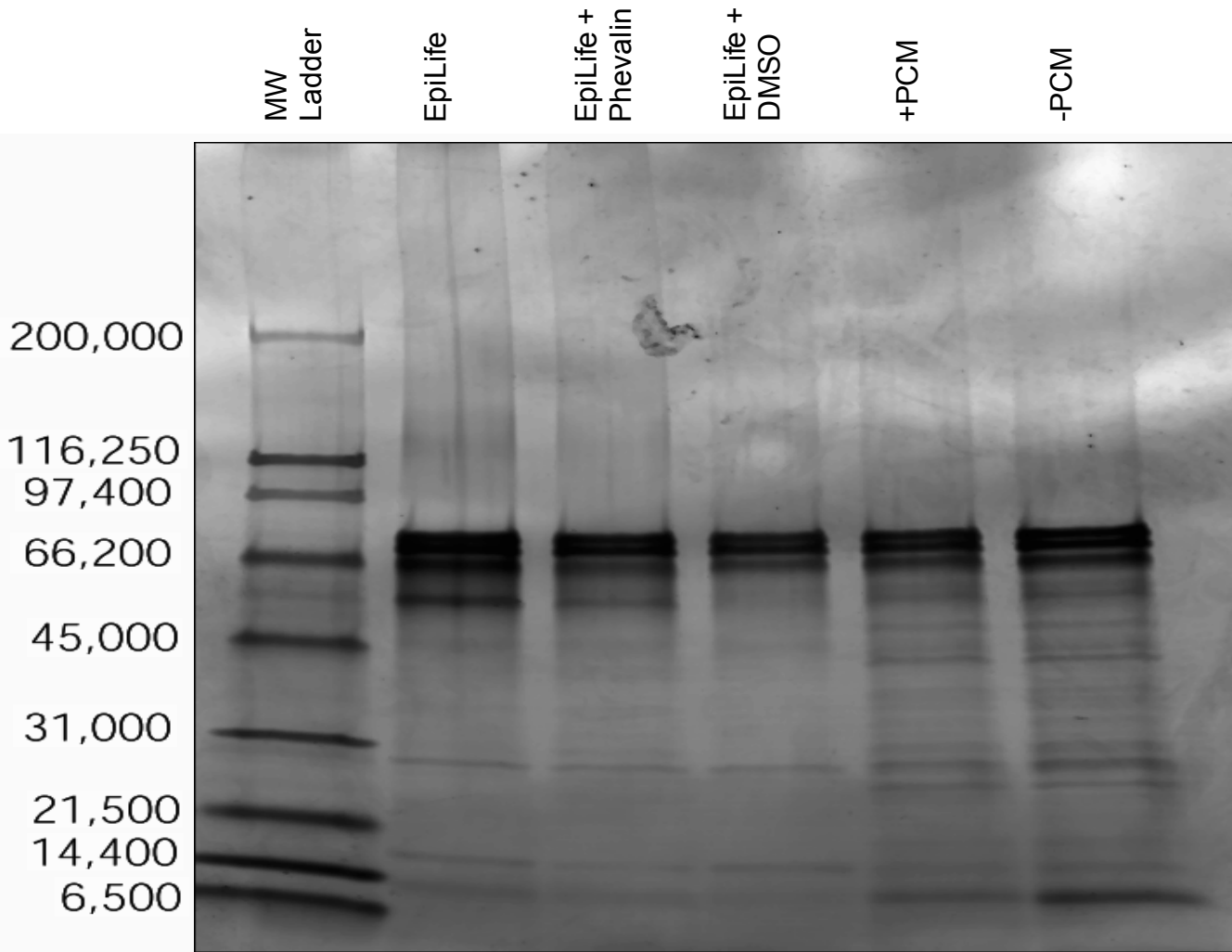

Supplement: Figure S2 — 1D SDS-PAGE analysis of extracellular proteins in growth medium control and +PCM and −PCM. No obvious differences were apparent between +PCM and −PCM. Gel stained with Sypro Ruby with a lower detection limit of 0.25–1 ng. (PDF) [file pone.0040973.s002.pdf]
