# Supplementary material for: Phevalin (aureusimine B)Production by Staphylococcus aureus Biofilm and Impacts on Human Keratinocyte Gene Expression
Source: PLoS One. 2012 Jul 13;7(7):e40973. doi: 10.1371/journal.pone.0040973 (PMC3396627; doi:10.1371/journal.pone.0040973)
Supplement: Table S1 — Genes significantly (p<0.05) regulated at least 2 fold in HKs treated with +PCM, −PCM, or BCM, relative to controls. (PDF) [file pone.0040973.s003.pdf]

Table S1. Genes significantly (p<0.05) regulated at least 2 fold in HKs treated with +PCM, -PCM, or BCM, relative to controls.

Gene Symbol	BCM v Control	+PCM v Control	-PCM v Control
ATF3	49.9	30.8	11.7
AVPI1	15.8	19.9	6.1
TSC22D3	7.1	19.2	17.3
C5orf13	8.9	15.4	16.1
HES1	85.6	14.8	4.7
PER1	4.4	13.8	9.1
H3F3B	2.0	13.1	6.7
FAM117A	3.2	12.7	11.1
CD55	15.8	11.2	5.1
DLX2	88.8	10.3	4.9
CD55	17.1	9.9	5.4
STC2	2.4	9.4	11.4
NAV2	1.7	9.3	8.4
PER1	4.6	9.1	10.2
CA12	4.0	9.1	5.7
DUSP1	154.7	8.5	3.2
C14orf147	1.5	8.4	6.5
VAV3	1.4	8.2	10.0
BLMH	3.8	8.1	6.9
ETS2	3.0	7.9	5.2
BCL6	8.2	7.9	8.6
KIAA0182	2.0	7.8	4.9
PIK3R3	4.0	7.7	6.6
PRKCA	1.7	7.5	5.3
CAMK2G	3.1	7.5	5.2
PRSS8	5.3	7.5	13.3
MTMR11	3.3	7.5	6.5
TGFBR3	2.3	7.3	3.8
C19orf42	2.5	7.2	5.2
BCL11A	2.2	7.0	5.7
MXI1	4.4	6.7	6.9
RGS3	1.3	6.7	4.4
STON1	1.3	6.5	8.0
CYP1A1	6.5	6.4	12.4
DLX4	2.6	6.3	7.3
LPHN1	2.6	6.2	5.3
PAPOLG	1.4	6.0	6.1
KRT16	6.2	5.9	6.8
FYN	2.2	5.8	5.9
KIAA0907	3.4	5.8	5.2
NFATC3	2.1	5.7	3.9
GAST	3.3	5.7	2.9
PCF11	1.1	5.7	2.8
NR4A2	20.0	5.7	2.2
DNM1	3.0	5.6	5.6
GSTA4	5.7	5.6	6.8
KRT1	5.2	5.6	7.3
FABP4	6.4	5.5	5.7
---	2.4	5.4	7.5
FAM8A1	2.3	5.4	6.1
TLE1	1.5	5.3	3.5
MEGF9	2.3	5.3	6.7
DFNB31	2.4	5.3	3.8
PNRC1	6.9	5.2	5.6
CDKN1A	6.2	5.2	4.1
JMJD6	1.3	5.2	2.1
CXCL14	3.6	5.2	4.6
FOS	325.9	5.2	2.0
HIST3H2A	3.4	5.2	4.5
EXD2	1.7	5.2	2.8
ALDOC	3.9	5.0	5.3
MMP1	9.9	5.0	3.8

ZNF516	1.1	4.9	4.2
COL1A1	1.9	4.9	3.3
EFNA3	1.8	4.9	5.3
SIK1	7.0	4.8	3.0
SLC7A8	2.2	4.8	3.8
DFNB31	2.0	4.7	2.9
STXBP1	2.9	4.7	4.8
CHN2	2.0	4.7	3.5
ADCY9	1.4	4.7	2.7
NR4A2	22.2	4.6	2.0
KLF9	5.5	4.6	4.4
MTSS1	2.9	4.6	5.1
PRR14	2.7	4.6	4.0
HBP1	2.3	4.6	4.1
LOC157562	1.7	4.6	5.5
AKR1C1	5.6	4.6	4.6
COL6A1	2.6	4.6	3.8
FOSB	138.3	4.5	1.4
---	3.1	4.5	4.8
LRRC20	2.0	4.4	3.5
TXNIP	1.2	4.4	4.5
ACSL1	2.4	4.4	6.0
CAMK2G	2.8	4.4	4.0
ERBB3	1.9	4.4	7.1
SOX4	2.0	4.4	3.2
MBOAT2	3.6	4.4	4.2
DNAJB6	2.8	4.4	3.4
MYCL1	1.9	4.3	7.5
MKKS	2.4	4.3	4.0
GPRC5C	3.2	4.3	3.2
MAP2K5	2.5	4.3	3.0
MAFB	4.6	4.2	6.1
EFNA1	6.8	4.1	2.7
WDR19	1.7	4.1	4.0
ZNF580	1.6	4.1	3.1
IQCE	1.4	4.1	3.4
DDIT3	2.3	4.1	5.9
GOLPH3L	2.1	4.0	4.4
PRSS3	3.5	4.0	2.8
HIST2H2BE	3.1	4.0	2.6
PLA2G4A	5.5	4.0	4.5
CCBL1	3.2	4.0	3.9
TCF4	1.6	4.0	3.4
SMYD3	2.7	4.0	3.6
PTPN6	2.1	4.0	3.1
---	1.2	4.0	4.6
SLC6A15	3.5	4.0	4.0
HECA	2.4	3.9	3.5
KDM3A	3.1	3.9	2.6
BTG2	3.5	3.9	5.7
CA12	2.7	3.9	3.8
DDX52	2.9	3.9	3.1
AKR1C2	4.5	3.9	3.8
LPHN1	1.8	3.9	3.6
PRR14	2.2	3.9	2.9
MAST3	1.5	3.9	3.5
C1orf63	3.8	3.9	1.9
VAMP2	-1.0	3.9	2.8
GEM	157.9	3.9	2.1
NR4A2	28.2	3.8	1.8
ZMYM3	1.4	3.8	3.0
ZFC3H1	-1.0	3.8	3.9
CKB	3.0	3.8	3.6
WDR8	2.0	3.8	3.8
PBX3	1.8	3.8	3.0
ELOVL4	2.6	3.8	4.9
FZD2	1.9	3.8	4.4

CSTF3	2.3	3.8	5.0
KIAA0368	1.5	3.8	3.4
CTSK	1.9	3.8	3.2
GALNT14	3.3	3.8	2.6
LMO4	2.7	3.8	3.9
TLE1	1.6	3.7	3.7
HSPBAP1	1.7	3.7	3.6
RERE	1.1	3.7	2.8
C1orf21	1.8	3.7	2.7
BNIP3	4.1	3.7	3.3
ARID5B	4.8	3.7	4.2
NT5DC2	3.1	3.7	3.7
NFIL3	2.0	3.7	3.4
ING1	5.9	3.7	2.5
RHOB	8.5	3.7	4.8
CSNK2A2	1.8	3.7	4.0
GABARAPL1 /// GABARAPL3	1.5	3.7	2.7
SV2A	2.0	3.7	3.5
PBXIP1	1.4	3.7	3.2
SLC7A8	2.1	3.6	3.5
CLTCL1	1.7	3.6	2.4
---	1.8	3.6	2.6
MYLIP	1.4	3.6	4.9
KCNMA1	3.0	3.6	3.0
BANP	2.0	3.6	2.8
B3GNT1	1.0	3.6	4.5
SLC25A12	2.6	3.6	3.1
SNX6	2.8	3.6	3.0
HSBP1	3.3	3.6	2.7
SLCO4A1	4.2	3.6	2.6
NDRG1	2.9	3.6	2.8
BFSP1	2.1	3.5	2.8
KRT6B	3.4	3.5	2.7
RBM4B	1.2	3.5	2.5
LARP6	1.0	3.5	3.5
NR2F6	1.5	3.5	2.5
FXYD3	2.7	3.5	4.3
HOXA5	1.6	3.5	2.8
MAP2K5	3.9	3.5	4.9
UBR5	2.1	3.5	3.7
USP21	1.9	3.5	3.0
PMS1	7.7	3.5	4.5
RIPK2	2.7	3.5	3.5
MYST1	2.0	3.4	3.0
LPHN2	2.3	3.4	4.6
LPHN1	1.8	3.4	3.5
ACLY	2.3	3.4	3.4
RCOR3	6.7	3.4	2.3
WBP2	1.5	3.4	2.8
HES1	48.0	3.4	1.7
HOXC6	1.7	3.4	3.7
PRSS3	3.0	3.4	2.4
RBM9	1.4	3.4	2.8
STK38L	1.2	3.4	3.9
ZNF238	1.1	3.4	6.0
FAM189B	2.1	3.4	3.0
DAPK2	1.5	3.4	2.3
ANKRD10	5.6	3.3	3.7
KIF3C	1.8	3.3	2.3
ACYP1	1.7	3.3	2.7
PEX6	1.9	3.3	4.6
ABHD4	1.4	3.3	3.1
HAGH	2.6	3.3	3.4
SLC30A1	2.1	3.3	2.8
CTNNBIP1	2.2	3.3	2.8
TMCC2	1.2	3.3	3.2
DLEU1	2.2	3.3	3.4

ULK1	1.9	3.3	5.7
ATP2B4	1.6	3.3	2.9
HOXA9	1.1	3.3	2.4
RSAD1	1.5	3.2	3.2
PTPRZ1	4.7	3.2	4.3
RNF130	1.8	3.2	3.5
EPAS1	2.1	3.2	3.0
AKR1C3	2.7	3.2	5.0
SYNGR1	2.5	3.2	2.5
FUCA1	1.1	3.2	3.2
FOXO1	1.7	3.2	3.0
PPP1R15A	34.0	3.2	2.8
ANKRD28	1.5	3.2	2.2
ARG2	2.5	3.2	2.9
FGFR4	3.1	3.1	3.2
ITM2C	2.2	3.1	2.6
IL1RN	2.4	3.1	2.2
ELL3 /// SERINC4	2.5	3.1	4.9
ERP44	1.2	3.1	2.9
INSR	1.8	3.1	2.6
PPM1A	1.9	3.1	2.5
LEPRE1	1.6	3.1	2.1
PRDM2	1.3	3.1	2.0
MIA3	2.0	3.1	2.5
FANCE	2.4	3.1	2.6
AGPAT1	1.4	3.1	3.1
BTG1	3.2	3.1	3.3
---	1.6	3.1	2.3
TBC1D9	1.6	3.1	3.6
GLS	2.5	3.1	3.6
ELL3 /// SERINC4	2.8	3.1	8.7
FAF1	2.7	3.0	2.5
ZNF250	1.3	3.0	2.7
MAN2A1	2.0	3.0	3.0
MTMR4	2.6	3.0	4.4
LOC151162 /// MGAT5	1.4	3.0	2.4
SREBF1	2.1	3.0	3.4
RABGAP1L	1.7	3.0	2.8
B3GAT3	1.7	3.0	2.9
EPB41L4B	2.1	3.0	4.0
SLC26A6	2.4	3.0	2.4
CA12	2.4	3.0	3.0
EGLN1	2.5	3.0	2.9
B3GAT3	1.8	3.0	2.6
GDPD5	1.8	3.0	2.5
RABGGTA	3.3	3.0	2.2
TTC13	4.1	3.0	4.2
FAM59A	2.6	3.0	2.6
HSPA4L	1.9	3.0	2.4
FAM46A	1.8	3.0	2.4
MAP4	1.4	3.0	2.3
LMO4	2.0	3.0	2.7
EIF2S2	1.2	3.0	2.2
CLMN	1.8	3.0	3.7
RALB	2.8	3.0	2.7
VPS16	1.8	3.0	1.9
MPHOSPH9	2.0	2.9	3.6
KCNG1	1.6	2.9	3.1
PIK3C2B	1.1	2.9	4.0
PHF20	-1.4	2.9	2.7
MAP1LC3B	3.5	2.9	1.9
EPHB3	3.0	2.9	3.3
BRD3	1.3	2.9	3.6
H2AFJ	1.0	2.9	3.4
PDE8A	2.4	2.9	2.2
CDKN2C	1.0	2.9	3.5
C14orf139	1.5	2.9	3.4

AKR1C1	4.0	2.9	3.4
AGPAT1	1.3	2.9	3.0
CXXC1	2.2	2.9	2.1
NDRG3	2.0	2.9	2.2
TFRC	3.0	2.9	2.6
RFX5	1.9	2.9	3.7
PINK1	1.3	2.9	2.0
---	2.5	2.9	2.7
EFNA5	1.6	2.9	2.7
CNNM4	1.6	2.9	2.5
FGFR3	2.2	2.8	2.5
BNIP3	3.2	2.8	2.7
MIPEP	2.8	2.8	2.4
MARCKSL1	2.1	2.8	2.6
MTMR4	1.8	2.8	2.7
SCAPER	2.4	2.8	1.9
LGTN	3.2	2.8	2.7
SH3GLB1	1.8	2.8	2.7
TMEM57	2.3	2.8	3.0
NME4	2.4	2.8	2.4
C12orf47	-1.0	2.8	2.9
MAP3K8	7.3	2.8	2.1
HTRA1	1.9	2.8	2.5
DST	2.4	2.8	3.4
COX16	1.7	2.8	1.8
CHKB-CPT1B /// CPT1B	4.6	2.8	3.4
DOCK4	1.8	2.8	3.0
CHAC1	1.1	2.8	14.4
ZMYM4	2.5	2.8	2.6
SLC25A36	2.0	2.8	2.6
FZD10	1.7	2.8	3.8
NR4A1	15.1	2.8	1.3
INSIG1	1.1	2.8	3.0
PLAGL1	2.9	2.7	2.6
SEMA3F	1.8	2.7	2.4
ABHD6	1.8	2.7	2.3
VEGFA	13.2	2.7	2.4
HOMER2	1.8	2.7	2.1
---	1.5	2.7	2.3
SC4MOL	1.6	2.7	2.5
SNHG3	-1.8	2.7	2.5
CLCN3	3.0	2.7	4.5
CRYAB	1.8	2.7	2.6
C11orf75	1.7	2.7	3.7
PDE8A	3.6	2.7	3.7
PPP1R15A	40.3	2.7	2.5
N4BP2L2	2.1	2.7	2.8
PHF7	2.0	2.7	2.6
DVL2	1.6	2.7	2.2
LGALS8	2.9	2.7	4.2
MVK	2.5	2.7	3.2
TMEM231	1.6	2.7	2.3
SYNGR3	1.1	2.7	3.1
RAD54B	2.5	2.7	2.8
SIX2	1.6	2.7	2.3
HSD17B7	2.3	2.7	2.4
CA12	1.8	2.7	2.9
C17orf59	2.2	2.7	5.5
TFAP2A	1.4	2.7	2.5
S100A4	2.2	2.7	2.1
C20orf111	4.8	2.7	1.6
AFF1	1.7	2.7	3.0
CXXC1	2.7	2.6	2.9
TTLL5	1.6	2.6	2.2
CACNB3	1.7	2.6	2.2
SDC3	1.1	2.6	2.3
POLA1	1.6	2.6	2.2

GRHL2	1.8	2.6	3.1
RSL1D1	2.7	2.6	1.9
SOS1	4.6	2.6	1.8
WIPF1	2.4	2.6	3.8
RNASE4	1.3	2.6	4.2
PLS1	3.3	2.6	3.1
MPP1	1.7	2.6	2.9
GSTM3	2.3	2.6	2.3
TMEM187	1.6	2.6	2.9
GRK5	1.6	2.6	2.6
LPIN1	1.4	2.6	2.1
UBE4B	1.9	2.6	2.3
POMZP3 /// ZP3	2.3	2.6	2.0
ECM1	1.8	2.6	2.3
METTL3	1.9	2.6	2.0
SNX27	1.3	2.6	2.2
SYNPO	1.3	2.6	2.6
TNNT1	2.3	2.6	2.2
TARBP1	3.0	2.6	1.9
FAM168A	1.4	2.6	2.2
ANG	1.5	2.6	3.1
PTP4A1	3.8	2.6	1.3
RALGPS1	2.0	2.6	2.8
RIMS3	1.4	2.6	3.2
KIAA0182	1.7	2.6	2.8
PFN2	1.7	2.5	3.1
CDK19	1.3	2.5	4.2
IP6K2	2.3	2.5	1.7
TULP3	1.7	2.5	2.4
SNCA	1.3	2.5	2.3
TCF4	1.2	2.5	2.3
TECPR2	1.2	2.5	2.1
ARID3A	1.2	2.5	2.2
IFT20	1.8	2.5	2.3
ATP6V0A1	1.5	2.5	2.2
CNTN1	3.8	2.5	2.7
SERTAD2	1.2	2.5	2.5
ING1	1.6	2.5	1.9
CLK3	1.8	2.5	2.4
PKIG	2.4	2.5	2.6
EDEM2	1.8	2.5	2.3
LGALS8	1.7	2.5	2.7
DLG3	1.3	2.5	2.4
ITSN2	1.5	2.5	2.4
SERPINI1	1.9	2.5	4.9
AP1M2	1.6	2.5	2.2
PELI1	3.8	2.5	2.4
VPS8	1.5	2.5	2.3
PAIP2B	1.5	2.5	4.3
C1orf106	1.8	2.5	2.6
PHF21A	2.8	2.5	2.6
POMZP3	2.2	2.5	2.2
CDK12	1.2	2.5	2.9
LPIN1	1.8	2.5	2.1
TCF4	1.0	2.5	2.1
TYRO3	2.1	2.5	2.0
MICB	1.8	2.5	3.2
PTOV1	2.1	2.5	3.0
SLC27A5	2.2	2.5	2.3
MICA /// MICB	2.6	2.5	2.8
ID3	7.1	2.4	2.6
LMBRD1	3.0	2.4	2.5
GPAA1	2.3	2.4	2.2
TCFL5	1.8	2.4	2.5
AQP3	3.0	2.4	2.8
ZBTB5	-1.1	2.4	2.3
MEN1	1.1	2.4	2.4

KIAA0513	1.1	2.4	3.2
NPC1	1.5	2.4	2.1
CSNK2A1	2.4	2.4	2.3
MEIS1	1.3	2.4	2.5
RCAN1	1.7	2.4	2.7
SCML1	2.3	2.4	3.0
HOXA9	1.4	2.4	2.1
ZNF185	1.4	2.4	2.5
IMPA2	1.5	2.4	2.7
FXR2	1.4	2.4	2.6
PEX6	1.9	2.4	3.3
PRSS16	2.4	2.4	2.6
PRKD2	1.6	2.4	2.7
DENND1A	3.0	2.4	2.1
TRPC1	1.9	2.4	2.8
FRAT2	1.4	2.4	2.9
HEG1	1.2	2.4	3.0
PPOX	1.5	2.4	2.4
NMB	2.0	2.4	2.0
DENND3	1.1	2.4	2.0
MVK	2.7	2.4	3.6
MSL3	2.2	2.4	2.0
ANKZF1	1.2	2.4	3.0
MKRN1	1.6	2.4	2.2
BAHCC1	1.2	2.3	2.7
GTPBP3	1.6	2.3	2.1
CRYL1	1.9	2.3	2.4
KCTD13	1.6	2.3	2.2
ATG5	-1.1	2.3	2.4
ZNF184	1.4	2.3	3.1
DDIT4	2.2	2.3	3.1
C1orf107	2.7	2.3	2.1
BAT2L1	1.1	2.3	2.2
TMEM180	2.3	2.3	3.1
CASP9	1.3	2.3	1.8
DOPEY1	1.3	2.3	2.6
CHKB-CPT1B /// CPT1B	2.9	2.3	2.0
ZCCHC24	1.2	2.3	3.1
BCORL1	1.2	2.3	2.2
C9orf116	1.8	2.3	2.1
NPRL2	2.0	2.3	2.2
ADCK2	2.4	2.3	2.6
PTEN /// PTENP1	2.0	2.3	2.0
ZCCHC11	2.0	2.3	3.5
C16orf45	1.5	2.3	2.6
TBC1D17	1.4	2.3	2.2
NARF	1.7	2.3	2.1
KDELR3	2.4	2.3	2.1
YPEL5	1.6	2.3	2.1
MLF1	2.2	2.3	2.1
DOK4	-1.2	2.3	2.0
PHF1	1.6	2.3	2.2
CPEB3	1.8	2.3	2.3
RER1	1.3	2.3	2.2
PTEN	2.1	2.3	2.0
ING1	4.1	2.2	1.7
---	2.1	2.2	1.9
SULF1	1.5	2.2	2.5
IDI1	1.4	2.2	2.1
VGLL4	1.5	2.2	2.2
P4HA2	1.7	2.2	2.3
PLAGL1	1.9	2.2	2.1
ANKS1A	-1.2	2.2	2.0
GYG1	3.0	2.2	2.8
FOXJ2	1.2	2.2	2.2
RAP1GAP	1.6	2.2	2.1
IQCK	3.0	2.2	2.4

C8orf55	2.6	2.2	2.4
EFNB3	1.9	2.2	2.6
RANGRF	2.1	2.2	2.2
SLC1A4	1.9	2.2	3.0
RTN3	1.8	2.2	2.4
B4GALT3	2.4	2.2	2.0
ZNF394	1.9	2.2	1.3
SOX12	1.5	2.2	3.7
MZF1	1.9	2.2	2.1
TMEM161A	2.1	2.2	1.9
FARP2	1.2	2.2	2.2
FBXL15	1.8	2.2	3.6
STK3	2.4	2.2	2.1
NEU1	4.5	2.2	3.7
C14orf93	1.4	2.2	2.5
TMEM47	2.2	2.2	3.8
TNK2	1.5	2.2	2.0
CLN5	2.1	2.2	2.6
IDI1	1.4	2.2	2.1
C1orf66	1.3	2.2	2.3
TSPYL4	-1.1	2.2	3.9
C14orf132	1.1	2.2	2.4
SLC9A6	1.2	2.2	2.5
EZH1	1.1	2.2	2.1
HLTF	3.5	2.2	3.9
SESN1	-1.0	2.2	2.4
POR	2.2	2.2	2.6
SNPH	1.2	2.2	2.4
REL	3.7	2.2	2.3
GLRX	-1.2	2.2	2.4
VAT1	1.4	2.2	2.1
CYHR1	1.3	2.2	2.2
TBPL1	2.0	2.2	1.7
DNMT3B	1.6	2.2	3.1
PASK	1.6	2.1	2.2
PRSS1 /// PRSS2 /// PRSS3	2.5	2.1	2.0
ATG9A	1.2	2.1	2.7
ACLY	1.8	2.1	2.8
CYLD	1.1	2.1	2.3
IDE	2.4	2.1	1.6
P4HA1	3.3	2.1	2.5
SGCE	1.3	2.1	2.1
SPRR1A	2.0	2.1	2.1
BHLHE40	2.7	2.1	1.3
ATP2B4	1.4	2.1	2.3
ODC1	2.5	2.1	1.7
HABP4	-1.2	2.1	1.6
PBX1	1.1	2.1	2.9
VAV3	1.3	2.1	2.8
GPI	1.9	2.1	1.9
DCAF10	1.0	2.1	2.0
IPO13	1.3	2.1	2.1
SPON2	1.4	2.1	2.6
NFATC1	1.0	2.1	2.2
NUPR1	1.2	2.1	3.4
AKR1C2	4.8	2.1	4.0
CYP39A1	1.5	2.1	3.2
CCNG2	1.5	2.1	3.0
SQLE	1.5	2.1	2.3
IDH2	2.2	2.1	2.3
CDC42BPA	1.1	2.1	2.2
C9orf9	1.6	2.1	2.0
CD320	2.7	2.1	2.0
CASK	2.5	2.1	2.5
CLEC2B	2.7	2.1	1.6
UST	1.1	2.1	2.4
SAT1	2.8	2.1	1.8

AVIL	1.5	2.0	2.1
EIF1	1.9	2.0	1.7
VAMP2	-1.2	2.0	2.5
TDRD3	3.2	2.0	1.8
PLK1S1	2.2	2.0	2.5
SNN	1.2	2.0	3.1
ZNF304	-2.0	2.0	1.2
PPM1D	5.8	2.0	2.4
FYN	1.3	2.0	2.5
NBL1	1.6	2.0	2.1
METTL2B	1.1	2.0	2.1
LOC91316	2.7	2.0	1.7
UNC119	1.6	2.0	2.9
TSC22D1	2.2	2.0	1.7
WNT5A	1.8	2.0	2.9
ELMO3	2.1	2.0	1.5
GALNT10	1.4	2.0	2.3
MZF1	2.1	2.0	1.8
RNF103	4.0	2.0	2.2
TMEM97	2.0	2.0	1.8
DVL2	1.5	2.0	2.3
TCEA2	1.5	2.0	2.5
RNASE4	1.0	2.0	3.4
H1F0	1.2	2.0	2.6
RFC4	1.5	2.0	2.0
DIXDC1	1.3	2.0	4.2
MAPRE2	2.3	2.0	1.9
---	1.0	2.0	2.8
SCAP	1.4	2.0	2.0
SIRT4	1.4	2.0	2.2
CEP63	2.0	2.0	1.8
NBN	4.2	1.9	1.7
BCAT2	1.6	1.9	2.2
C18orf8	2.2	1.9	2.0
RHOT1	1.7	1.9	2.3
ADA	2.3	1.9	2.2
GPR19	1.8	1.9	2.3
ASAP3	1.2	1.9	2.3
IDE	2.0	1.9	1.5
C2orf68	-1.1	1.9	2.6
---	2.2	1.9	2.6
ACSL1	1.8	1.9	2.3
ROBO1	1.5	1.9	2.0
HRSP12	1.8	1.9	2.1
ZC3H12A	6.3	1.9	1.2
ZNF204P	1.5	1.9	3.9
SLC1A4	1.3	1.9	2.2
SULF1	1.5	1.9	2.1
SQLE	2.5	1.9	5.0
LOC100286937 /// LOC100287164 /// RASA4	2.2	1.9	3.4
TSC22D3	2.7	1.9	14.6
SLC1A4	1.6	1.9	2.2
MLLT11	1.6	1.9	2.3
DNAJB2	1.4	1.9	2.2
PIGV	1.4	1.9	2.2
ECSIT	2.5	1.9	1.8
BMP2	4.6	1.9	2.4
DSG1	4.4	1.9	4.7
ABHD6	1.9	1.9	2.2
DGAT1	3.1	1.9	2.4
DCLK1	2.5	1.9	2.3
C2orf68	-1.1	1.9	2.1
TWIST1	1.2	1.9	2.4
WDR67	1.9	1.9	3.0
BOP1 /// LOC727967	2.1	1.9	1.7
WIPI1	-1.4	1.9	3.8
MMD	1.1	1.9	2.6

VIM	2.2	1.9	2.1
FAM190B	1.6	1.9	2.5
SAT1	3.0	1.9	1.4
SLC25A12	1.9	1.8	3.2
GULP1	1.9	1.8	2.3
EXTL2	1.5	1.8	2.4
IQCG	2.1	1.8	2.2
40792	1.2	1.8	2.2
S100PBP	-1.8	1.8	2.2
INPP4A	1.4	1.8	2.1
NQO2	1.5	1.8	2.3
KLHL24	5.8	1.8	1.7
TFEB	-1.1	1.8	2.2
CEP57	1.9	1.8	2.4
CBFA2T2	1.1	1.8	2.1
STX3	3.2	1.8	1.1
PLAG1	1.2	1.8	3.1
EGR1	130.2	1.8	1.2
CCDC28A	1.0	1.8	2.3
NXF1	2.6	1.8	1.6
POT1	2.2	1.8	2.4
PLCD1	1.3	1.8	2.1
PIK3CD	1.2	1.8	2.2
MAF	1.9	1.8	2.5
LDLR	2.1	1.8	2.1
PSPC1	2.7	1.8	1.8
C6orf48	1.7	1.8	2.0
IGFBP6	2.1	1.8	1.9
L2HGDH	1.3	1.8	2.1
IL22RA1	1.4	1.8	2.1
IRF2	1.2	1.8	2.1
SCAPER	2.0	1.8	1.8
GADD45G	3.6	1.8	1.9
CYP51A1	4.3	1.8	2.6
MGEA5	-1.4	1.8	2.1
DBP	1.4	1.8	4.0
RAB7L1	2.4	1.8	6.2
HADH	2.1	1.8	2.0
INSIG1	1.4	1.8	4.1
MEF2D	2.2	1.8	1.5
GUSB	2.2	1.8	1.4
PIM1	2.0	1.7	1.7
SLC9A3R1	1.3	1.7	2.1
CA12	2.2	1.7	2.4
ETS2	3.1	1.7	1.8
GPAA1	2.0	1.7	2.1
RAB26	1.1	1.7	2.2
MPP2	1.6	1.7	2.0
MARK1	2.7	1.7	1.1
SMCR7L	-1.8	1.7	2.3
PCYT2	2.4	1.7	2.1
JUNB	11.5	1.7	1.0
GOLGA8B	3.5	1.7	2.4
KRCC1	2.2	1.7	2.5
RAB22A	1.1	1.7	2.1
INSIG2	-1.1	1.7	2.9
TRIM52	1.4	1.7	3.1
TIPARP	2.8	1.7	2.0
PPP2R3A	2.0	1.7	1.9
PCYOX1L	1.4	1.7	2.1
TRPC1	1.5	1.7	2.8
ZNF34	9.6	1.7	1.3
MOSPD2	1.3	1.7	2.5
RPP25	1.4	1.7	2.5
BTG1	1.9	1.7	2.0
SLC39A8	2.7	1.7	2.4
SOX4	1.1	1.7	2.6

AKAP8L	3.0	1.7	3.3
RPL28	2.1	1.7	1.5
FBXL6	2.4	1.7	2.0
PTEN	2.1	1.7	1.7
KIAA0232	-2.3	1.6	1.3
ATF2	3.5	1.6	1.4
FAM69A	1.2	1.6	2.1
HERPUD1	1.7	1.6	2.1
IFT81	1.1	1.6	2.2
ANKRD5	2.8	1.6	1.9
IDE	2.1	1.6	1.5
---	5.4	1.6	1.1
PREPL	2.8	1.6	1.8
VEGFA	15.6	1.6	1.2
CNOT8	1.2	1.6	2.8
TSTA3	2.2	1.6	1.6
EGR1	45.6	1.6	2.0
STX10	2.2	1.6	2.4
IER2	4.3	1.6	1.1
TNFSF9	3.9	1.6	3.0
DUSP10	12.3	1.6	1.1
PDSS2	2.1	1.6	1.8
GHR	1.6	1.6	2.4
CXCR7	2.2	1.6	1.8
PRKD2	1.6	1.6	2.7
SKAP2	1.5	1.6	2.0
GPHN	2.3	1.6	2.3
CXorf57	1.8	1.6	2.5
ZNF692	3.0	1.6	1.5
PTPN4	2.1	1.6	1.9
UCKL1	2.2	1.6	1.1
KLF4	5.6	1.5	1.7
CHMP1B	3.9	1.5	1.5
SLC2A3	2.3	1.5	-1.1
FAM69A	1.3	1.5	2.1
---	1.4	1.5	2.1
CGRRF1	1.5	1.5	2.4
CREM	2.0	1.5	2.3
TTBK2	-1.3	1.5	2.3
CHKA	2.5	1.5	2.5
SNX17	2.2	1.5	1.8
TMEM48	2.2	1.5	1.4
FAM13A	1.9	1.5	2.8
MAN1A1	3.6	1.5	1.5
GAA	2.2	1.5	3.4
---	3.2	1.5	1.1
NINJ1	1.2	1.5	2.6
AUTS2	-2.0	1.5	1.2
PBXIP1	1.5	1.5	3.4
EPOR	1.2	1.5	2.1
FGF18	5.9	1.5	1.0
KLHL24	5.2	1.4	1.8
NEAT1	7.5	1.4	1.3
LAPTM4B	2.1	1.4	2.4
SMAD7	3.3	1.4	-1.2
SPAG9	2.1	1.4	1.3
RAD51C	1.7	1.4	2.5
IL6	6.2	1.4	-1.0
TGIF1	2.3	1.4	1.1
DHCR7	2.0	1.4	2.4
FERMT2	3.2	1.4	1.3
WSB1	2.1	1.4	1.2
RNF216	-1.1	1.4	2.2
PPP1R10	2.7	1.4	2.1
PSPH	2.0	1.4	3.0
DUSP1	5.0	1.4	1.1
KDM5B	2.1	1.4	1.7

CEBPA	1.3	1.4	3.3
ARIH1	4.2	1.4	1.2
MAF	1.6	1.4	2.2
FGF18	8.4	1.4	1.1
CLASP2	3.4	1.4	1.8
C2orf3	3.4	1.4	1.5
RNF19B	2.9	1.4	1.2
ZNF434	-2.3	1.4	1.4
KPNA5	2.7	1.4	1.2
VEGFA	5.7	1.4	2.0
CYP1B1	1.6	1.4	2.6
SERTAD3	2.9	1.4	1.5
AKAP9	2.2	1.3	1.5
PVRL3	2.5	1.3	2.2
H1FX	2.0	1.3	3.8
FAM172A	1.8	1.3	2.3
EGR2	14.5	1.3	-1.0
GALNT6	-3.1	1.3	1.1
NDRG3	2.1	1.3	2.0
CEBPB	2.0	1.3	1.7
CTH	1.9	1.3	3.1
SEC31A	3.0	1.3	2.6
SPTAN1	-2.1	1.3	1.1
RB1CC1	1.8	1.3	2.8
ANKRD26	2.4	1.3	1.1
MMP3	3.6	1.3	-1.0
GLA	2.0	1.3	1.3
NECAB3	2.2	1.3	1.3
ZFP36	10.9	1.3	-1.3
C9orf95	1.4	1.3	2.4
RIPK4	3.5	1.3	1.3
SPTAN1	-2.1	1.3	1.3
TRIP11	2.3	1.3	1.3
VCPIP1	2.2	1.3	1.2
GPS1	2.2	1.3	1.5
LPPR2	1.9	1.3	2.1
CALM3	-2.0	1.3	1.1
PTGS2	13.9	1.3	1.2
OVOL1	3.2	1.2	1.3
EIF1AX	-2.4	1.2	1.2
SH2B2	1.1	1.2	2.2
SMCR7L	-2.8	1.2	1.0
MSH3	2.1	1.2	1.3
HIST1H2BG	3.7	1.2	1.4
VEGFA	12.6	1.2	1.2
FGF18	7.3	1.2	1.0
---	40.3	1.2	-1.1
ANKRA2	-1.1	1.2	3.1
CXCL3	2.8	1.2	1.0
CYP1B1	1.4	1.2	2.9
MYST3	-2.6	1.2	-1.2
BHLHE40	4.1	1.2	-1.0
CUL4B	2.2	1.2	1.3
PEX2	5.0	1.2	2.4
ZNF750	2.9	1.2	1.1
C11orf17 /// NUAK2	3.1	1.2	-1.1
MLEC	-2.1	1.2	-1.0
ZBTB25	2.2	1.2	1.3
ID2	6.9	1.2	1.2
RLF	2.0	1.2	-1.0
PCMTD2	1.4	1.2	2.3
IL8	17.9	1.2	1.0
CAMSAP1	-2.2	1.1	1.1
IDS	2.2	1.1	1.1
PSIP1	2.0	1.1	2.1
RNF19B	3.1	1.1	1.3
---	2.3	1.1	1.1

SLC38A2	2.4	1.1	1.2
GNA13	5.2	1.1	1.0
SLC25A10	2.4	1.1	1.6
RIT1	5.2	1.1	1.4
POT1	1.8	1.1	2.1
EFNA4	1.2	1.1	2.4
SLC38A2	2.4	1.1	1.2
RALB	3.4	1.1	2.0
FKBPL	2.9	1.1	-1.0
TTLL5	3.0	1.1	-1.0
ABL2	3.8	1.1	-1.3
PAFAH1B1	-2.9	1.1	1.1
GADD45B	2.7	1.1	-1.2
IL8	89.6	1.1	-1.3
SUN1	2.4	1.1	1.2
PSEN1	2.1	1.1	1.7
GNB2L1	4.7	1.1	1.0
GOLGA3	-2.6	1.1	-1.2
PURA	-2.2	1.1	1.1
NUP50	-2.9	1.0	-1.3
ZFAND5	2.6	1.0	1.1
GLUL	2.4	1.0	1.2
MAP4K5	2.0	1.0	1.3
CHD8	-2.0	1.0	-1.1
HSPC157	1.3	1.0	2.1
NEDD4L	2.2	1.0	-1.2
IL1A	18.2	1.0	1.5
---	1.1	1.0	-2.2
PNP	2.3	1.0	-1.4
FEM1B	-2.5	1.0	1.4
PDGFA	-2.1	1.0	-1.4
HIST1H2AE	2.7	1.0	1.0
CCNL1	5.4	1.0	-1.1
GTF2E1	-2.1	1.0	1.0
BMP2	3.1	1.0	1.6
DNAJB4	1.9	1.0	3.9
FBXL18	-2.0	1.0	1.2
MVD	7.1	1.0	4.5
SEC16A	-2.2	-1.0	-1.2
TPRA1	1.5	-1.0	2.9
DSC2	2.8	-1.0	1.4
HBEGF	3.3	-1.0	-1.4
ZC3H4	-2.0	-1.0	-1.2
SORL1	-2.3	-1.0	-1.1
GPR172A	1.8	-1.0	2.3
LARP1	-2.4	-1.0	1.1
IER5	2.2	-1.0	-1.2
ATXN1	-2.2	-1.0	1.1
APPBP2	2.0	-1.0	1.4
FAM108B1	2.6	-1.1	1.2
MGEA5	3.6	-1.1	1.1
GJB3	1.4	-1.1	-3.0
OLFML2A	-3.3	-1.1	-1.1
CXCL2	6.6	-1.1	-1.4
JUN	4.3	-1.1	-1.2
---	4.6	-1.1	1.2
PDCD4	1.7	-1.1	2.2
RIN3	-2.1	-1.1	-1.4
GOLGA6L4 /// PML	-2.3	-1.1	-2.1
SPATA2	-2.1	-1.1	-1.1
ID2	11.3	-1.1	1.3
ALG13	2.2	-1.1	-1.1
GJB3	1.5	-1.1	-3.6
EIF1	3.5	-1.1	-1.1
KLF4	3.1	-1.1	1.1
C5orf54	1.2	-1.1	2.6
JUN	2.5	-1.1	-1.5

MEF2A	-2.4	-1.1	1.1
HBEGF	6.3	-1.1	-1.5
NFKBIA	3.2	-1.2	-1.1
GLUD2	2.6	-1.2	2.0
WSB1	2.7	-1.2	1.1
EPHB3	3.2	-1.2	3.1
ARIH1	2.7	-1.2	-1.2
MAFF	5.0	-1.2	-2.5
TRIB2	-2.2	-1.2	-1.1
PPL	-2.1	-1.2	-1.5
IL1B	4.3	-1.2	-1.6
CITED2	4.9	-1.2	1.0
TP63	3.0	-1.2	1.0
IL11	7.8	-1.2	-1.2
SVIL	-2.2	-1.2	-1.4
CDC27	2.2	-1.2	1.0
ADM	2.2	-1.2	-1.3
NEBL	-2.3	-1.2	-1.7
DUSP7	-1.8	-1.2	-3.0
SH2D3A	1.2	-1.2	-2.0
TM2D3	-2.4	-1.2	1.1
ZYX	-2.2	-1.2	-1.3
CDC25A	-1.1	-1.2	-2.1
C12orf41	-2.1	-1.2	-1.1
IL1B	4.0	-1.2	-1.4
LTBR	-1.6	-1.2	-2.1
SYNRG	-2.1	-1.2	-1.2
ABCC10	-1.5	-1.3	-2.0
CITED2	15.4	-1.3	1.4
IKBKB	-2.1	-1.3	-1.2
SLC20A1	1.4	-1.3	-2.6
C3	-2.0	-1.3	-1.8
LTB4R	1.7	-1.3	2.6
IER3	3.9	-1.3	-2.2
MLEC	-2.5	-1.3	-1.3
CXCL1	8.4	-1.3	-1.4
ARL14	6.9	-1.3	-1.2
HNRNPH3	-2.3	-1.3	-1.1
L1CAM	-3.7	-1.3	-1.2
DUSP10	8.6	-1.3	-1.6
MTA1	1.2	-1.3	2.1
RARA	-1.7	-1.3	-2.3
TNFAIP3	32.9	-1.3	-2.0
IL1RL1	6.3	-1.3	-1.8
GTSE1	-3.1	-1.3	-1.7
FARSA	2.0	-1.3	1.2
VDR	-2.1	-1.3	-1.1
TP63	-1.5	-1.3	-2.0
LGALS3BP	-2.3	-1.4	-1.7
ASNS	-4.0	-1.4	1.5
INTS7	2.1	-1.4	1.2
TAPBP	-2.1	-1.4	-1.1
NOL12 /// TRIOBP	-2.2	-1.4	-1.8
KIFC1	-3.5	-1.4	-1.8
ST14	-2.6	-1.4	-1.3
SPRY4	1.8	-1.4	-2.0
TRIO	-2.4	-1.4	-1.9
CBFB	-2.2	-1.4	-1.2
---	-2.2	-1.4	-1.0
ATAD2	2.5	-1.4	1.8
PNN	-3.1	-1.4	-1.1
NUP160	-1.3	-1.4	-2.2
PCDH1	-2.1	-1.4	-1.4
CDC27	2.2	-1.4	1.1
NOL6	-1.8	-1.4	-2.4
RIOK3	5.0	-1.4	1.2
NOTCH2	-2.1	-1.4	-1.2

MAFF	4.0	-1.4	-5.0
ZNF12	-3.5	-1.4	1.0
BAT2L1	-3.0	-1.5	-1.2
ATF7IP	3.3	-1.5	-1.8
HIRA	-3.2	-1.5	-1.6
INTS5	-3.8	-1.5	-1.2
SUN2	-2.2	-1.5	-1.6
PTRH2	-1.9	-1.5	-2.5
ZFYVE21	-2.9	-1.5	1.8
CHSY1	-2.1	-1.5	-1.5
ELL2	2.3	-1.5	-2.0
NDUFAF4	-1.4	-1.5	-2.3
TROVE2	-2.1	-1.5	1.1
SEC24A	2.2	-1.5	-1.7
GDI2	2.0	-1.5	1.5
CHMP6	-1.2	-1.5	-2.2
PAIP1	-3.0	-1.5	-1.4
SLPI	-1.8	-1.5	-2.4
FLNB	-2.5	-1.5	-2.4
C16orf53	-1.5	-1.5	-2.1
SAMD4A	-2.1	-1.5	-1.6
PTK2B	-3.0	-1.6	-2.3
VEGFC	-1.2	-1.6	-2.4
BAT1	2.1	-1.6	-1.5
APP	-3.6	-1.6	-1.7
TMCC1	2.3	-1.6	-2.2
TTC3	-3.3	-1.6	-1.0
C17orf91	4.6	-1.6	-1.6
DDX18	-2.3	-1.6	-1.7
MMP10	3.8	-1.6	-2.2
KDM2A	-2.7	-1.6	-1.5
IL4R	-2.0	-1.6	-2.2
EHD1	-1.6	-1.6	-2.3
KLF7	-1.1	-1.6	-2.4
EMG1	-1.7	-1.6	-2.0
SNAPC4	-3.4	-1.6	-2.0
JUN	1.3	-1.6	-2.6
ZC3HAV1	-1.0	-1.6	-2.5
KHNYN	-1.6	-1.6	-3.2
C9orf114	-1.4	-1.6	-2.4
DRG2	2.1	-1.6	1.7
ZCCHC2	-2.3	-1.6	-1.5
JUND	2.8	-1.6	2.6
SERPINE1	1.1	-1.6	-2.3
GTSE1	-2.2	-1.6	-1.9
ERLIN2	-2.5	-1.7	-1.1
CDC42EP3	-2.0	-1.7	-1.6
C14orf1	1.3	-1.7	2.3
PMEPA1	-1.8	-1.7	-2.7
UPK3B	-2.3	-1.7	-1.8
EPHA2	10.6	-1.7	-4.3
C10orf2	-1.7	-1.7	-3.7
TRIOBP	-2.2	-1.7	-2.1
CTNND1	-2.1	-1.7	-1.4
STK10	-2.2	-1.7	-1.8
SLC25A15	-2.2	-1.7	-2.0
RND3	2.5	-1.7	-1.8
TMEM40	-1.2	-1.7	-3.0
ITGA6	2.1	-1.7	1.6
NEFL	-2.4	-1.7	-1.7
EFEMP1	-2.1	-1.7	-1.8
RAB40B	-2.2	-1.7	1.0
NRG1	-1.2	-1.7	-2.1
MICAL2	-3.9	-1.7	-2.8
POLR1C	-1.6	-1.7	-2.4
EHD1	-1.6	-1.8	-2.4
SFRS7	6.1	-1.8	-1.8

RAB5C	-2.0	-1.8	-1.7
ETNK1	2.1	-1.8	-1.8
CARD10	-1.4	-1.8	-2.7
PFKFB3	-1.3	-1.8	-2.3
ST14	-2.5	-1.8	-1.9
ADRB2	4.6	-1.8	-3.7
CEP72	-1.6	-1.8	-2.3
THBD	-1.9	-1.8	-2.2
HADHA	-3.2	-1.8	-1.6
SAV1	-2.7	-1.8	-1.6
PLAU	1.4	-1.8	-6.6
MYO1E	-2.1	-1.8	-2.1
SLC7A6	-1.8	-1.8	-2.2
DKK3	-2.3	-1.8	-1.9
TAF5L	-2.3	-1.8	-2.3
C17orf63	-2.1	-1.9	-1.1
CDK16	-2.1	-1.9	-1.6
SCHIP1	1.2	-1.9	-2.7
SGK1	2.7	-1.9	-1.4
VDR	-2.6	-1.9	-1.3
TTF1	-2.2	-1.9	-1.3
AGR2	-2.1	-1.9	-1.5
IMPA1	2.0	-1.9	-1.0
ESRP2	-1.7	-1.9	-2.4
SMARCC1	-2.3	-1.9	-1.2
PALLD	-2.3	-1.9	-1.1
BCAR3	-1.1	-1.9	-5.6
PLEKHG3	-1.2	-1.9	-2.5
PPIC	-2.0	-1.9	-1.4
SAMD4A	-1.5	-1.9	-2.5
RRM2	-1.2	-1.9	-2.7
HOXD11	-1.9	-1.9	-2.0
TACSTD2	-1.7	-1.9	-2.4
CHAF1A	-4.7	-1.9	-1.8
ADAM19	-2.4	-1.9	-2.4
DHDDS	-2.0	-1.9	-1.9
CDCA3	-2.4	-2.0	-2.1
NPAT	-2.1	-2.0	-1.4
SYNJ2BP	-2.6	-2.0	-1.6
---	-2.3	-2.0	-2.0
SRRM2	-2.1	-2.0	-2.0
DUSP3	-1.6	-2.0	-2.5
TRIM14	-2.6	-2.0	-2.0
MVK	4.2	-2.0	4.4
NUFIP1	-2.3	-2.0	-2.2
ASXL1	-3.0	-2.0	-2.1
FEZ2	-2.0	-2.0	-2.1
TMEM127	-2.4	-2.0	-2.3
ASB9	-2.3	-2.0	-2.3
TCEB3	-2.9	-2.0	-1.9
PTGES	-2.5	-2.0	-2.8
PPIF	-1.2	-2.0	-2.8
NAT10	-2.3	-2.0	-2.7
HR	-2.9	-2.0	-2.9
40795	-2.3	-2.0	-1.7
C17orf71	-3.9	-2.0	-1.9
EIF5	-1.7	-2.0	-1.8
SYNCRIP	-1.9	-2.0	-2.4
EHD1	-1.5	-2.0	-2.5
NDST1	-3.3	-2.0	-2.1
TPM1	-2.2	-2.0	-2.1
TOE1	-1.2	-2.0	-2.7
PARVA	-2.1	-2.0	-2.0
HOOK2	-2.4	-2.0	-2.1
PRKRIR	-2.6	-2.1	-1.9
BCAS2	-1.2	-2.1	-1.8
PAIP1	-3.5	-2.1	-1.7

UBTF	-2.1	-2.1	-1.5
TUSC3	-2.1	-2.1	-1.5
AMACR /// C1QTNF3	-1.4	-2.1	-2.0
MCFD2	-2.2	-2.1	-2.2
PANX1	-1.5	-2.1	-2.4
IGFBP3	-2.2	-2.1	-2.4
RCHY1	-2.1	-2.1	-1.8
PRSS23	-2.6	-2.1	-2.2
DUSP5	13.8	-2.1	-3.2
PWP2	-1.7	-2.1	-2.1
PTK6	-2.1	-2.1	-3.0
FASTKD3	-1.5	-2.1	-2.2
WDR3	-1.6	-2.1	-2.3
PXDN	-2.8	-2.1	-2.1
MGAT2	-2.9	-2.1	-1.3
IGFBP3	-2.8	-2.1	-2.7
PPP1R12A	-3.4	-2.1	-2.4
CBX6	-2.4	-2.1	-1.1
CASP1	-2.0	-2.1	-2.0
ZGPAT	-1.8	-2.1	-2.6
HUS1	-1.7	-2.1	-2.2
TRIM16	-1.3	-2.1	-2.2
NOP56	-1.4	-2.1	-2.6
FAM64A	-2.1	-2.1	-1.7
TRIB1	2.3	-2.1	-3.0
C12orf4	-1.0	-2.1	-2.4
ITGB8	1.2	-2.1	-2.3
PAPOLA	2.8	-2.2	1.6
AGPAT2	1.0	-2.2	-1.2
TMF1	-3.9	-2.2	-2.7
EIF2S3	3.0	-2.2	2.4
RRS1	-1.3	-2.2	-2.3
MAPRE1	2.2	-2.2	-1.4
COIL	-2.2	-2.2	-2.0
DOLK	-2.3	-2.2	-1.4
RRP15	-1.3	-2.2	-2.4
TSSC4	-1.9	-2.2	-2.5
ERAP1	-1.9	-2.2	-2.0
ID1	20.0	-2.2	-3.0
RCN1	-3.1	-2.2	-2.9
FBXO21	-3.9	-2.2	-1.3
DHFR	-1.3	-2.2	-2.3
NUTF2	1.1	-2.2	1.1
MAZ	1.4	-2.2	1.3
TPM1	-2.5	-2.2	-1.9
IPW	-2.4	-2.2	-1.4
CCNF	-2.8	-2.2	-1.7
FHL2	-1.6	-2.2	-2.5
EGR3	10.2	-2.2	-2.0
RRBP1	-4.3	-2.2	-2.5
FAS	-1.9	-2.2	-2.3
WWTR1	1.2	-2.2	-3.5
TBC1D2	-1.6	-2.2	-2.4
EMP1	-1.5	-2.2	-2.7
ENDOD1	-2.2	-2.2	-1.7
MLH3	-2.0	-2.2	-2.3
HJURP	-2.6	-2.2	-1.7
SEC14L1	-3.1	-2.3	-2.5
GRPEL1	-1.2	-2.3	-2.3
LMNB1	-2.6	-2.3	-2.1
GOSR2	-1.6	-2.3	-2.6
GOSR1	-2.6	-2.3	-2.0
JRKL	-3.7	-2.3	1.0
MAK16	-2.5	-2.3	-2.8
NACC2	-4.6	-2.3	-1.6
SYNCRIP	-1.6	-2.3	-2.5
BTBD7	-1.2	-2.3	-2.6

ASB6	-1.7	-2.3	-2.3
JAG1	-1.1	-2.3	-2.7
ZNF226	-1.8	-2.3	-2.4
NEFL	-2.9	-2.3	-2.0
AK3L1	-1.2	-2.3	-2.1
CCDC94	-1.9	-2.3	-2.6
EMP1	-1.7	-2.3	-3.2
RFC3	-1.9	-2.4	-2.4
FOXD1	-1.3	-2.4	-3.2
AKAP13	-3.2	-2.4	-2.2
KBTBD4	-1.8	-2.4	-2.4
CENPA	-3.1	-2.4	-1.7
PKP1	-1.6	-2.4	-2.2
UPF1	-1.7	-2.4	-1.5
PDLIM4	-2.1	-2.4	-1.6
C6orf120	-2.4	-2.4	-1.4
C13orf15	-3.5	-2.4	-5.7
MAP3K7	-2.1	-2.4	-2.1
GRWD1	-1.4	-2.4	-4.1
ZFP36L2	1.0	-2.4	-2.3
JAG1	-1.0	-2.4	-2.8
CYR61	2.6	-2.4	-3.2
PUS3	-3.0	-2.4	-2.5
HSPA8	-1.1	-2.4	-2.2
TM7SF3	-2.0	-2.4	-1.5
ITGA2	-2.5	-2.5	-2.4
RPP38	-1.8	-2.5	-2.3
UBXN4	-3.1	-2.5	-2.0
AP1S1	-2.0	-2.5	-1.8
SLC35D1	-2.1	-2.5	-1.9
C11orf24	-1.8	-2.5	-2.4
NID2	-2.6	-2.5	-2.7
PLK4	-1.9	-2.5	-2.2
MSL1	1.2	-2.5	-2.3
SLC43A3	-2.1	-2.5	-3.3
DNM2	1.2	-2.5	1.1
TUSC3	-2.5	-2.5	-1.7
SPRED2	1.3	-2.5	-2.5
TJP1	-2.6	-2.5	-1.7
GEMIN6	-2.2	-2.5	-2.3
PPP2R1B	-1.2	-2.5	-2.2
E2F3	-1.7	-2.5	-2.4
FBXL14	-2.3	-2.5	-1.2
ERGIC2	-2.1	-2.5	-1.9
WWTR1	-1.7	-2.6	-2.1
WDR43	-1.7	-2.6	-3.1
FAM111A	-3.9	-2.6	-2.7
PAPOLA	-2.9	-2.6	-2.0
INPP1	-2.7	-2.6	-2.5
RBM28	-1.6	-2.6	-3.2
PTK7	-2.7	-2.6	-2.0
RGS20	-2.3	-2.6	-2.6
AASDHPPT	-1.6	-2.6	-2.1
PLAU	2.6	-2.6	-3.8
NUPL1	2.1	-2.6	-3.4
MICAL2	-3.5	-2.6	-3.4
RBMS1	-1.8	-2.6	-2.2
NR2F2	-2.2	-2.6	-2.0
SH3TC1	-2.0	-2.6	-2.5
RASAL2	-1.6	-2.6	-2.9
KLHL7	-1.2	-2.6	-2.3
WASL	-2.6	-2.6	-1.9
FUS	-1.5	-2.6	-2.0
KIF14	-2.8	-2.6	-1.4
TGFA	-1.9	-2.6	-3.6
NRAS	-1.1	-2.6	-2.2
ANKRD11	-3.0	-2.7	-1.5

PPRC1	-1.6	-2.7	-3.6
ETNK1	1.1	-2.7	-2.8
RNF25	-2.3	-2.7	-1.9
MGAT4B	1.5	-2.7	2.4
BCLAF1	1.3	-2.7	-2.5
NAV3	-1.4	-2.7	-8.3
DNASE2	-2.3	-2.7	-1.3
LSM5	-2.1	-2.7	-1.7
PVR	-1.8	-2.7	-3.5
HNRNPUL2	-2.1	-2.7	-2.5
DCAF7	-3.5	-2.7	-2.0
KLK5	-1.3	-2.7	-1.3
THBS1	-2.0	-2.8	-3.3
SERPINB6	-2.4	-2.8	-2.6
NET1	-1.5	-2.8	-2.9
KIF20A	-2.7	-2.8	-1.8
CBX5	-2.3	-2.8	-2.9
MDFI	-2.3	-2.8	-2.9
GPN2	-2.2	-2.8	-2.7
AGPAT5	-1.9	-2.8	-2.3
CD2BP2	-1.8	-2.8	-3.1
NUP153	-1.3	-2.8	-3.5
ACBD3	-5.0	-2.8	-2.6
TNFAIP3	6.4	-2.8	-4.4
DNAJB1	4.6	-2.8	-2.7
NMU	-3.9	-2.8	-2.2
---	-3.2	-2.8	-2.2
EPM2AIP1	-1.4	-2.8	-2.2
C1orf175 /// TTC4	1.2	-2.9	-2.2
CFLAR	-4.1	-2.9	-3.4
ZFP36L2	-1.3	-2.9	-3.8
FAS	-2.4	-2.9	-1.7
NRG1	1.3	-2.9	-5.3
PTGER2	-3.1	-2.9	-3.1
DUSP11	-2.5	-2.9	-2.2
M6PR	-2.9	-2.9	-1.6
ATF5	-1.2	-2.9	-2.8
NUCB1	-1.0	-2.9	2.1
GNG11	-2.7	-2.9	-3.4
NQO1	-2.1	-2.9	-1.9
SMAD3	-1.9	-2.9	-3.2
KIAA0020	-1.5	-2.9	-3.5
PHLDA3	-1.7	-3.0	-1.3
BMP2K	-1.0	-3.0	-3.3
EXOSC4	-1.2	-3.0	-2.2
ALDH7A1	-2.2	-3.0	-2.2
GALNT7	-2.0	-3.0	-2.0
ACVR1B	-2.4	-3.0	-1.9
SPAG1	-1.1	-3.0	-2.2
RBM12	-1.5	-3.0	-5.1
GLG1	-2.3	-3.0	-1.9
COG8 /// PDF	-1.9	-3.0	-4.2
C13orf34	-3.2	-3.0	-2.3
MMP14	-3.0	-3.0	-3.6
EIF5	-2.4	-3.0	-1.9
KHSRP	-3.1	-3.0	-2.2
ZBTB24	-2.5	-3.0	-2.9
TMEM185B	-1.9	-3.0	-4.1
KLHL9	-2.5	-3.1	-1.8
MKI67	-2.7	-3.1	-2.7
TFAM	-3.4	-3.1	-3.4
MCAT	-1.3	-3.1	-1.9
SNRNP35	-2.0	-3.1	-2.5
FASTKD2	-1.9	-3.1	-2.6
IVNS1ABP	-2.0	-3.1	-3.2
RHOBTB3	-2.9	-3.1	-1.6
CTSZ	-3.0	-3.1	-2.6

TMEM158	-4.0	-3.1	-3.9
GRAMD3	-1.2	-3.1	-2.2
ENDOD1	-1.9	-3.1	-2.6
CD3EAP	-2.1	-3.1	-4.4
FADS3	-1.6	-3.2	1.0
DDX43	1.0	-3.2	-2.6
ARHGAP29	-1.7	-3.2	-2.6
C15orf39	-2.2	-3.2	-1.9
TFAM	-3.8	-3.2	-3.2
RIN2	-1.7	-3.2	-2.8
ELOVL1	-1.4	-3.2	-1.5
ARPP19	-1.6	-3.3	-2.3
TP53I3	-3.3	-3.3	-3.9
EFEMP1	-2.8	-3.3	-2.4
CFLAR	-4.3	-3.3	-3.6
SLC31A2	1.0	-3.3	-2.9
GTF2H4	-2.5	-3.3	-2.7
C12orf5	-1.7	-3.3	-2.7
CENPA	-3.6	-3.3	-2.0
CNOT3	-1.2	-3.3	1.3
NFYA	-1.3	-3.3	-3.4
PCSK5	-4.5	-3.3	-3.2
PPIG	-2.5	-3.3	-2.9
LXN	-2.9	-3.4	-2.7
ZNF267	-5.8	-3.4	-2.9
PDLIM4	-2.7	-3.4	-3.1
DNAJB6 /// TMEM135	-1.3	-3.4	-2.2
CYTH2	-1.4	-3.4	-4.0
MTR	-2.1	-3.4	-3.4
WTAP	-1.2	-3.4	-3.1
CCND1	-2.5	-3.4	-3.7
NUP62CL	-3.1	-3.4	-3.0
UBN1	-3.9	-3.4	-2.0
GJB5	-1.4	-3.4	-7.0
UTP3	-2.2	-3.4	-2.3
GATC	-2.0	-3.4	-2.7
EREG	7.4	-3.5	-3.9
RBM15	-1.8	-3.5	-2.4
TIMM8A	-2.9	-3.5	-2.9
PPP2R1B	-3.0	-3.5	-3.0
GCLM	-2.6	-3.5	-2.4
SART3	-3.4	-3.5	-1.8
CHD4	-1.7	-3.6	-1.5
WSB2	-1.7	-3.6	-3.6
CRCP	-2.7	-3.6	-3.0
EHD1	-1.1	-3.6	-3.2
CLDN1	-1.3	-3.6	-4.8
CCNA1	1.2	-3.6	-7.0
B4GALT5	-2.0	-3.7	-2.6
IGF1R	-3.1	-3.7	-4.4
CCNA2	-3.4	-3.7	-2.4
GADD45B	2.5	-3.7	-1.9
LOC100133109	-2.0	-3.7	-3.1
CTGF	3.0	-3.7	-4.5
ING3	-1.5	-3.7	-3.1
SOLH	-2.0	-3.8	-1.5
CSTF2T	-2.9	-3.8	-3.3
MMP14	-2.6	-3.8	-2.9
RRBP1	-6.0	-3.8	-3.9
TCP11L1	-2.4	-3.8	-3.1
GMFB	-2.0	-3.8	-2.7
STAT1	-2.0	-3.8	-2.2
CYR61	3.6	-3.8	-4.4
CSTF1	-2.1	-3.8	-2.3
TAGLN2	-1.2	-3.9	-1.4
MAVS	-1.7	-3.9	-2.7
SMAGP	-2.9	-3.9	-4.8

C1orf163	-2.9	-3.9	-3.4
CDK12	-2.5	-3.9	-4.9
ADORA2B	-1.6	-3.9	-3.9
FADD	-2.4	-3.9	-2.4
DDI2 /// RSC1A1	-2.4	-3.9	-4.0
ISG15	-4.2	-4.0	-3.8
CD164	-1.9	-4.0	-3.7
C7orf49	-2.3	-4.0	-3.1
TFAM	-1.8	-4.0	-2.2
NRG1	1.6	-4.0	-8.0
HPCAL1	1.3	-4.0	1.4
TDG	-1.0	-4.0	-2.7
PTGER4	-1.0	-4.0	-3.7
RUNX1	-1.9	-4.1	-6.6
OGDH	-2.0	-4.1	-1.5
PAWR	-1.0	-4.1	-3.3
ILF3	-2.9	-4.1	-2.3
KIAA1609	-5.1	-4.1	-2.9
CENPN	-1.7	-4.1	-3.4
ISOC2	-2.7	-4.1	-3.1
ESF1	-2.1	-4.1	-2.8
SOCS6	-1.9	-4.1	-2.6
AMD1	2.2	-4.1	-3.9
MRPS18A	-1.3	-4.2	-1.9
NRG1	-2.7	-4.2	-4.6
CSTF1	-2.2	-4.2	-2.3
EHBP1L1	1.1	-4.2	1.6
HNRNPC	-1.8	-4.2	-1.3
ZBTB1	-1.8	-4.2	-3.0
PNO1	-1.3	-4.2	-3.3
IRS1	-1.9	-4.2	-3.9
BRD2	-1.2	-4.2	-2.6
KRR1	-1.6	-4.2	-2.6
TSPAN1	-2.7	-4.2	-3.0
TPM1	-3.0	-4.2	-2.4
C17orf42	-1.8	-4.3	-2.5
BAT2	-4.9	-4.3	-3.4
ARL4C	-2.0	-4.3	-4.6
MPDU1	1.0	-4.3	-1.4
AMIGO2	-2.1	-4.3	-7.4
WTAP	-1.5	-4.3	-3.3
MTAP	-1.7	-4.4	-2.4
PHLDA1	-1.1	-4.4	-4.5
DKK3	-2.2	-4.4	-4.3
ZFR	-2.9	-4.4	-4.5
MAT2A	-3.9	-4.4	-3.5
RBM25	-2.2	-4.4	-3.3
DCBLD2	-2.3	-4.5	-3.6
EIF2C2	-2.5	-4.5	-5.8
TMX4	-2.9	-4.5	-2.5
KIAA1609	-5.3	-4.5	-3.3
SCNN1A	-4.9	-4.6	-4.9
BIN3	-3.9	-4.6	-3.2
SLC25A32	-1.2	-4.6	-2.5
MAP2K2	3.0	-4.6	2.6
DDX28	-2.7	-4.6	-2.5
CDC42BPA	-2.6	-4.7	-3.1
WDR73	-3.0	-4.7	-3.4
PPP3R1	-3.5	-4.7	-3.1
MCM9	-1.8	-4.7	-3.7
DYRK2	-4.1	-4.7	-2.7
VANGL1	-4.7	-4.7	-3.2
GABPB1	-2.4	-4.8	-6.0
PEX16	-2.2	-4.8	-3.1
DYRK2	-5.7	-4.8	-2.5
DUSP4	-1.1	-4.9	-10.4
PDS5A	-3.4	-4.9	-4.0

ARL5A	-2.8	-5.0	-2.9
FST	-2.2	-5.0	-13.4
POM121 /// POM121C	-2.1	-5.0	-2.1
FJX1	-2.4	-5.1	-4.6
PDLIM2	-3.5	-5.1	-4.3
GEMIN4	-2.9	-5.1	-4.5
SMC3	-2.5	-5.2	-1.6
METTL1	-2.9	-5.2	-8.1
SLC7A1	-4.9	-5.2	-4.1
TAGLN	-3.9	-5.3	-6.2
CREB1	-1.9	-5.3	-3.6
RAB5C	-3.1	-5.3	-2.4
MYC	1.6	-5.4	-11.8
KBTBD4 /// PTPMT1	-2.8	-5.4	-4.4
FOXE1	-1.9	-5.4	-3.9
KMO	-5.2	-5.5	-6.6
LIMS1	-2.6	-5.5	-2.5
TOP2A	-2.5	-5.5	-2.2
NOP56	-1.9	-5.6	-5.4
NUP98	-1.7	-5.6	-6.9
MTAP	-2.5	-5.6	-3.4
SCEL	-2.8	-5.6	-4.6
SYNJ2	-5.0	-5.6	-5.2
ALDH1A3	-7.2	-5.7	-5.1
LARP4	-2.1	-5.7	-5.2
KMO	-4.7	-5.7	-6.8
CALD1	-3.4	-5.7	-4.4
PTRF	-2.3	-5.9	-1.8
HPS6	-3.7	-5.9	-2.7
ATF5	-2.7	-6.0	-5.1
GPRC5A	-1.4	-6.1	-16.4
TMEM177	-3.0	-6.2	-2.3
LRRFIP1	-3.6	-6.3	-4.7
NRIP1	-9.3	-6.4	-7.6
ZFP36L1	-1.2	-6.4	-6.0
SYNJ2	-4.5	-6.4	-4.8
UBE2S	-1.8	-6.6	-2.1
AXL	-2.6	-6.7	-2.5
C1orf107	-2.0	-6.8	-5.2
ZBED5	-1.3	-6.8	-2.1
PLK2	-1.2	-6.8	-6.1
RIF1	-2.1	-6.9	-7.3
MED7	-4.7	-6.9	-3.6
C22orf29	-4.1	-6.9	-6.6
PLAC8	-7.6	-7.0	-8.0
CHST15	-2.7	-7.0	-4.2
MFAP5	-5.1	-7.1	-6.5
PCDH7	-6.0	-7.2	-7.3
---	-4.2	-7.2	-7.3
MRPL12	-2.5	-7.2	-3.7
TFPI2	-4.6	-7.2	-6.2
THBS1	-2.0	-7.3	-10.2
HSP90B1	-4.5	-7.3	-4.1
PPAN	-3.2	-7.3	-10.1
USP1	-6.0	-7.3	-4.5
F3	-1.4	-7.4	-14.2
AKAP2 /// PALM2-AKAP2	-6.1	-7.5	-6.4
C1orf107	-2.0	-7.5	-6.0
ADAMTS1	-1.5	-7.6	-8.8
ZNF365	-2.9	-7.7	-6.2
AP2B1	-4.9	-7.8	-4.0
BAT2	-7.7	-7.9	-4.2
DDA1	-1.8	-8.0	-3.4
MED7	-4.3	-8.2	-4.2
METAP2	-7.0	-8.3	-6.0
BAT2	-8.1	-8.4	-4.6
BCLAF1	-2.7	-8.5	-5.7

H2AFX	-2.4	-8.5	-2.8
TNS4	-4.0	-8.6	-8.5
CDK9	-1.5	-8.6	-3.1
ANP32A	-2.1	-8.6	-4.9
LCMT2	-2.2	-8.8	-3.5
CDC27	-2.2	-8.8	-4.0
ITGB4	2.4	-8.8	2.5
TPM4	-5.5	-8.8	-4.4
MFAP5	-8.1	-8.8	-10.0
SYNCRIP	-2.2	-8.8	-4.3
C12orf43	-3.5	-9.0	-7.5
UMPS	-3.0	-9.1	-4.5
NAT1	-4.1	-9.2	-5.2
C1orf116	-3.0	-9.5	-4.0
MFAP5	-8.9	-9.6	-13.6
CLDN7	-5.0	-9.9	-6.5
INHBA	1.0	-10.1	-15.2
MCL1	1.7	-10.1	-5.9
TAF15	-7.5	-10.2	-9.2
RBM16	-2.3	-10.2	-12.6
UMPS	-3.7	-10.2	-5.5
C14orf169	-5.5	-10.3	-8.9
GEMIN4	-3.9	-10.3	-8.0
DUSP4	1.0	-10.4	-28.5
NRG1	-6.4	-10.5	-13.9
ZFP36L1	-1.8	-10.9	-10.1
ARHGDIA	-1.6	-11.0	-3.6
C6orf62	-4.3	-11.2	-2.3
PTHLH	-2.3	-11.5	-84.0
PHLDA1	-3.5	-11.5	-13.7
SOX9	-1.6	-11.6	-23.4
CLPTM1	-2.6	-11.9	-1.9
NELF	-3.8	-12.2	-4.5
PTHLH	-2.3	-12.3	-79.6
ARL4C	-1.9	-12.4	-8.4
EZR	-8.6	-12.6	-12.3
DLEU2 /// DLEU2L	-8.7	-13.0	-11.1
TOP1	-7.0	-13.1	-13.8
PPIF	-1.2	-13.8	-4.7
DKK1	1.0	-13.9	-24.7
VASP	1.1	-14.4	-1.4
NAA15	-4.0	-15.0	-6.4
ARL4C	-2.1	-15.4	-11.3
SDHC	-3.1	-18.6	-2.0
AP1S1	-3.4	-18.9	-3.5
SON	-7.0	-19.8	-12.5
SERPINB2	-1.1	-20.0	-26.0
FLG	-13.9	-20.3	-19.9
HMGA2	-34.2	-23.1	-32.2
AP1S1	-5.6	-25.5	-6.0
DUSP6	1.1	-29.6	-62.1
SOX9	-1.4	-31.4	-46.2
CCND1	-12.4	-32.0	-28.4
FST	-2.0	-39.9	-111.4
FOSL1	-2.6	-41.2	-116.4
PHLDA1	-6.8	-45.4	-53.7
APOBEC3B	-29.8	-46.3	-33.9
ATP6V0E1	-2.5	-48.9	-11.2
PHLDA1	-1.8	-51.5	-58.3
AKAP12	-26.6	-60.3	-53.3
DUSP6	-1.1	-104.5	-97.5
DUSP6	-1.3	-129.3	-160.8
