# Supplementary material for: Phevalin (aureusimine B)Production by Staphylococcus aureus Biofilm and Impacts on Human Keratinocyte Gene Expression
Source: PLoS One. 2012 Jul 13;7(7):e40973. doi: 10.1371/journal.pone.0040973 (PMC3396627; doi:10.1371/journal.pone.0040973)
Supplement: Table S2 — Genes significantly (p<0.05) regulated at least 2 fold in HKs treated with +PCM relative to −PCM treated HKs. (PDF) [file pone.0040973.s004.pdf]

Table S2. Genes significantly ($p<0.05$) regulated at least 2 fold in HKs treated with +PCM relative to -PCM treated HKs.

Gene	Fold Change (+PCM/-PCM)
FOS	12.4
PTHLH	11.6
FOSB	10.0
EGR1	8.1
DUSP6	7.9
FST	5.4
FOSL1	5.2
ID1	5.0
TNFAIP3	4.9
EPHA2	4.7
PLAU	4.7
MYC	4.6
NAV3	4.6
DUSP5	4.5
GEM	4.2
DUSP1	4.0
PTP4A1	3.8
ADRB2	3.7
CD55	3.4
MAFF	3.4
HES1	3.3
BCAR3	3.3
DUSP4	3.2
SOX9	3.2
DLX2	3.2
SLC20A1	3.2
NRG1	3.1
C10orf2	3.1
INHBA	3.1
AVPI1	3.0
THBS1	3.0
CYR61	3.0
IER3	2.9
NR4A1	2.9
GJB3	2.8
NEDD4L	2.8
CSNK2A1	2.7
MMP10	2.7
HIST2H2BE	2.7

DUSP10	2.7
NR4A2	2.7
DKK1	2.7
SFRS7	2.7
SOS1	2.7
RUNX1	2.6
AMD1	2.6
BHLHE40	2.5
CITED2	2.5
KHNYN	2.5
PAPOLA	2.5
GPRC5A	2.5
TRIB1	2.5
RCOR3	2.4
KDM3A	2.4
TGFBR3	2.4
CCNA1	2.4
GJB5	2.4
NOP56	2.3
MAK16	2.3
MICAL2	2.3
RIOK3	2.2
IL1RN	2.2
UCKL1	2.2
IL1RL1	2.2
LMBRD1	2.2
RABGGTA	2.2
IP6K2	2.2
FLNB	2.1
USP21	2.1
PCF11	2.1
TMEM40	2.1
C13orf15	2.1
IER2	2.1
KIAA0020	2.1
FERMT2	2.1
PPP1R15A	2.1
ZFP36	2.1
JUNB	2.1
CTGF	2.1
ACSL1	2.1
JMJD6	2.1
SLC43A3	2.1

MAP3K8	2.1
ATF3	2.1
PSEN1	2.1
GPHN	2.1
CNTN1	2.1
PDE8A	2.1
ING1	2.0
H3F3B	2.0
HBEGF	2.0
ZNF394	2.0
REL	2.0
VEGFA	2.0
C11orf17 /// NUAK2	2.0
KLF7	2.0
C9orf95	-2.0
CYP1B1	-2.0
AP1S1	-2.0
DNAJB4	-2.0
HPS6	-2.0
LPPR2	-2.1
PRSS8	-2.1
ELOVL1	-2.1
ULK1	-2.1
ANKRA2	-2.1
DNM2	-2.1
MED7	-2.1
ASAP3	-2.1
C17orf59	-2.1
KLK5	-2.1
TAGLN2	-2.2
NUTF2	-2.2
MAZ	-2.2
ZNF204P	-2.2
EFNA3	-2.2
BTG2	-2.2
MYCL1	-2.2
UBE2S	-2.3
SOX12	-2.3
TMEM177	-2.3
KHSRP	-2.3
DBP	-2.3
VAMP2	-2.4
PTRF	-2.5

RNF216	-2.5
JRKL	-2.5
BAT2	-2.5
LCMT2	-2.6
FADS3	-2.6
RAB5C	-2.6
GPR172A	-2.6
EPHB3	-2.7
JUND	-2.7
GAA	-2.7
MPDU1	-2.7
AXL	-2.7
CEBPA	-2.7
PER1	-2.7
MVD	-2.7
LTB4R	-2.8
PBXIP1	-2.9
H1FX	-2.9
EFNA4	-2.9
SOLH	-3.0
MTA1	-3.0
NELF	-3.1
OGDH	-3.2
CLPTM1	-3.4
HNRNPC	-3.5
ITGB4	-3.8
EHBP1L1	-4.4
CNOT3	-4.8
TPRA1	-4.9
TSC22D3	-5.4
MGAT4B	-5.4
HPCAL1	-5.6
NUCB1	-6.3
MVK	-6.8
VASP	-7.3
SDHC	-7.7
CHAC1	-7.8
MAP2K2	-8.1
C6orf62	-12.2
