# Supplementary material for: Phevalin (aureusimine B)Production by Staphylococcus aureus Biofilm and Impacts on Human Keratinocyte Gene Expression
Source: PLoS One. 2012 Jul 13;7(7):e40973. doi: 10.1371/journal.pone.0040973 (PMC3396627; doi:10.1371/journal.pone.0040973)
Supplement: Table S3 — qPCR primer sequences. (PDF) [file pone.0040973.s005.pdf]

Table S3. qPCR primer sequences (5'-3')

Gene	Primer sequences (5'-3')	Ref
DUSP1	CGAGGCCATTGACTTCATAGA<br>CTGGCAGTGGACAAACACC	Nature Genetics 39, 503 - 512 (2007)
FOS	TGACTGATACACTCCAAGCGGA<br>CAGGTCATCAGGGATCTTGCA	Nature Genetics 39, 503 - 512 (2007)
ATF	AGCAGGCCCTTCCCATTC<br>AGTTGAGGCAAAGATG	Cell Death and Differentiation 15, 1472–1480 (2008)
